# Targeting the PI3K/AKT signaling pathway: an important molecular mechanism of herbal medicine in the treatment of MASLD/MASH

**DOI:** 10.3389/fnut.2025.1743899

**Published:** 2026-01-12

**Authors:** Shan Jiang, Li Zhai, Yingchun Shao, Fusheng Sun, Xuedong Liu

**Affiliations:** Qingdao Hospital, University of Health and Rehabilitation Sciences (Qingdao Municipal Hospital), Qingdao, China

**Keywords:** herbal medicine, MASH, MASLD, molecular mechanism, PI3K/AKT signaling

## Abstract

Metabolic dysfunction-associated steatotic liver disease (MASLD) was once known as non-alcoholic fatty liver disease (NAFLD). MASLD and its progressive form metabolic dysfunction-associated steatohepatitis (MASH) have become significant challenges in global public health with their incidence rates showing a persistent upward trend. MASLD is the result of multiple factors acting simultaneously. Multiple biochemical cycles and molecular pathways are implicated in the pathogenesis of MASLD/MASH, such as the phosphoinositide 3-kinase (PI3K)/ protein kinase B (AKT) signaling pathway. Natural products are considered a treasure for new drug discovery and are of great value to medicine. Natural products mediate their therapeutic effects on MASLD/MASH, at least in part, by modulating the PI3K/AKT signaling pathway. However, at present, there are relatively few systematic reviews and summaries in this field both domestically and internationally. This review systematically examines the molecular mechanisms through which the PI3K/AKT signaling pathway contributes to the pathogenesis of MASLD/MASH. Additionally, it places particular emphasis on the potential of natural compounds targeting this pathway as a preventive strategy against MASLD/MASH. It is evident that the PI3K/AKT signaling pathway plays an important role in the progression of MASLD/MASH. Natural products belonging to various classes, such as phenylpropanoids, flavonoids, terpenoids, and alkaloids, can alleviate MASLD/MASH and the mechanisms involve regulation of the PI3K/AKT signaling pathway. Herbal medicines hold significant research potential in modulating the PI3K/AKT signaling pathway to improve MASLD/MASH. An in-depth understanding of these pharmacological mechanisms will help design precise intervention strategies to effectively interrupt or reverse disease progression.

## Graphical abstract

**Figure:**
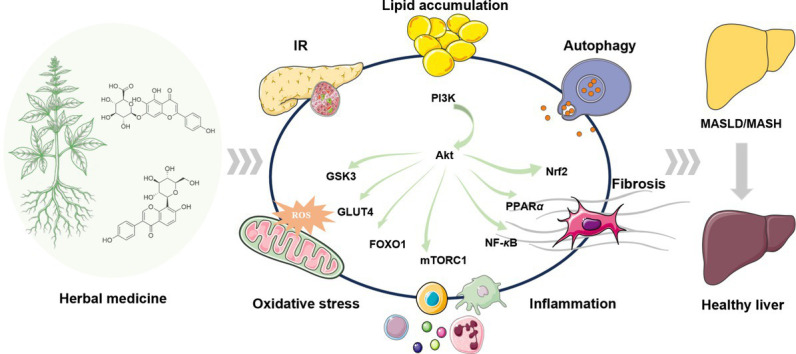

## 1 Introduction

Nonalcoholic fatty liver disease (NAFLD), now also termed metabolic dysfunction-associated steatotic liver disease (MASLD), is a hepatic disorder characterized by its strong associations with overweight, obesity, type 2 diabetes mellitus (T2DM), and metabolic syndrome (1). Without intervention, it may advance to nonalcoholic steatohepatitis (NASH) or metabolic dysfunction-associated steatohepatitis (MASH), a more severe inflammatory form, and potentially progress to hepatocellular carcinoma (2). Current epidemiological data suggest the global prevalence of MASLD is approximately 38%. It is anticipated that the rapid transition of the population to an aging society will further exacerbate this trend, potentially resulting in a sustained increase in both the incidence rate and mortality associated with the disease (3). The pathogenesis of MASLD is considered to begin with the “first hit” of simple steatosis, which is not enough to trigger inflammation and fibrosis. However, in the process of disease progression, a subsequent “second hit” is essential to exacerbate liver damage (4). Recently, the “multiple hits” hypothesis has been proposed to explain the underlying mechanism of the initiation and progression of MASLD. This hypothesis suggests that not only insulin resistance (IR) and oxidative stress, but also lipotoxicity, adipokines secreted by adipocytes, endotoxins [such as lipopolysaccharide (LPS)] released by gut microbiota, and endoplasmic reticulum (ER) stress have negative impacts simultaneously. Together, these factors propel the development of MASLD from simple fatty liver to MASH, liver fibrosis, and finally, end-stage liver diseases (5, 6). Additionally, it has been documented that environmental, nutritional, genetic and epigenetic factors are also associated with the pathophysiological basis of MASLD (7, 8).

Given that MASLD is a complex condition driven by the dysregulation of numerous interconnected biological pathways, the discovery of novel treatments remains a significant challenge. Against this backdrop, bioactive compounds derived from classic traditional Chinese medicine formulas provide promising approaches. These natural ingredients have clearly recorded hepatoprotective effects. Moreover, their effectiveness and safety have been proven through hundreds of years of clinical use. In recent years, the global acceptance of natural medicines has grown significantly, paralleling broader societal progress. Herbal remedies are now widely acknowledged in numerous countries as valuable complementary and alternative treatment options. Currently, a considerable amount of scientific evidence indicates that numerous natural herbal medicines have achieved positive results in the treatment of MASLD and MASH, such as *Psoralea corylifolia* L. (9), *Platycodon grandiflorum* (10). Moreover, certain monomeric compounds extracted from natural herbals exhibit considerable potential in the treatment of MASLD and MASH. Notable examples include alkaloids, flavonoids, terpenoids, and phenylpropanoids. Consequently, bioactive constituents derived from herbs represent a highly valuable repository for the discovery and development of therapeutic agents targeting MASLD and MASH (11).

The signaling pathway defined by phosphoinositide 3-kinase (PI3K) and protein kinase B (AKT) functions as a crucial signal transduction nexus. It effectively bridges extracellular stimuli with the intracellular signaling networks responsible for governing cellular responses (12). The PI3K/AKT signaling pathway belongs to a complex signaling axis, which is composed of a variety of upstream regulators and downstream effector molecules. Under physiological conditions, this signaling is triggered by growth factors, cytokines and hormones. Activated AKT subsequently phosphorylates its downstream targets (such as nuclear factor erythroid-2-related factor 2 (Nrf2), nuclear transcription factor-*κ*B (NF-*κ*B), forkhead box O (FOXO), glycogen synthase kinase-3 (GSK3), mechanistic target of rapamycin complex 1 (mTORC1), etc.) (13). These phosphorylated targets, in turn, activate multiple signaling pathways. In addition, interactions have been observed between the PI3K/AKT pathway and other key pathways, such as RAS/MAPK and JAK–STAT, contributing to an intricate signaling network (14). Increasing evidence has demonstrated that aberrant regulation of the PI3K/AKT signaling pathway within hepatocytes represents a frequent molecular alteration correlated with metabolic disturbances, including MASLD and MASH (15).

Here, we systematically sort out the core role of the PI3K/AKT signaling pathway in the key pathological processes involved in the progression of MASLD, including lipid metabolism, IR, inflammation, oxidative stress, etc. Given the pivotal role of the PI3K/AKT signaling pathway in the pathogenesis of MASLD/MASH, this review compiles natural compounds derived from medicinal herbs that exhibit protective effects by modulating this pathway. Consequently, this provides a theoretical foundation for future studies on herbal medicine-based treatments for MASLD/MASH and related metabolic disorders.

## 2 The involvement of the PI3K/AKT pathway in MASLD/MASH progression

The PI3K signaling pathway is commonly initiated by a diverse range of steroid hormones and various growth factors. Central to this pathway are the PI3K and AKT proteins, which serve as its key molecular components. According to their structural characteristics and substrate specificity, PI3Ks are systematically divided into three subclasses (I, II, and III). Class I PI3Ks represent the most extensively studied category of these kinases. Their structure is composed of a catalytic subunit, p110, and a regulatory subunit, p85 (16). Activated Class I PI3K phosphorylates PIP2 to generate PIP3, the activated form, on intracellular membranes. Subsequently, the signal propagation continues downstream, ultimately converging upon and activating the various AKT isoforms. It should be noted that this signaling cascade is strictly regulated by phosphatase and tensin homologue (PTEN), the primary antagonist of PI3K activity. PTEN counteracts PI3K signaling by catalyzing the dephosphorylation of PIP3, thereby regenerating its precursor, PIP2 (17). There are three isoforms of AKT protein (AKT1, AKT2, and AKT3), and its activation depends on two key phosphorylation events. The activation of AKT is a sequential process initiated by phosphoinositide-dependent protein kinase 1-mediated phosphorylation at the Thr308 residue within its kinase domain (18). This initial step is followed by a second phosphorylation event, which occurs at the Ser473 site located in the carboxy-terminal regulatory domain. Phosphorylation is catalyzed by the mTORC2 complex, whose own activity is dependent on PI3K signaling. The completion of this dual phosphorylation event is requisite for the full activation of AKT, enabling it to modulate its downstream effector pathways (17, 19, 20).

As the core regulatory network of cell growth, metabolism and survival, the PI3K/AKT signaling pathway exhibits pronounced functional duality. While the PI3K/AKT pathway is physiologically activated to regulate normal bodily functions, its activity is suppressed by excessive energy intake. Reactivation of this pathway under such pathological conditions can counteract obesity and IR. Conversely, dysregulation of PI3K, such as overexpression and mutation, contributes to the pathogenesis of multiple human diseases, including obesity and MASLD/MASH. Under such disease states, the pharmacological inhibition of PI3K constitutes a promising strategy to achieve therapeutic benefits with a favorable safety profile (19, 21).

**Figure 1:**
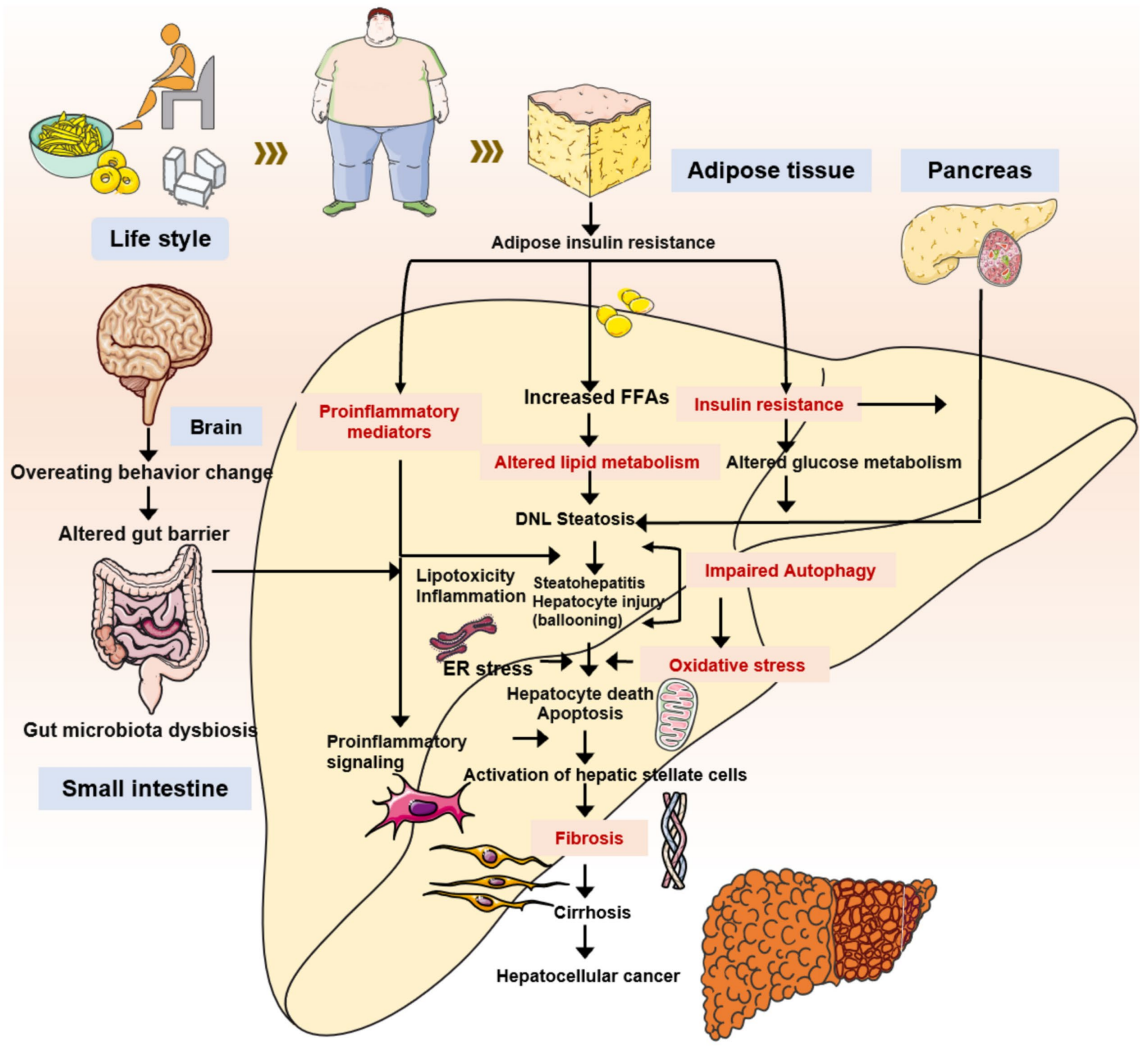
The primary mechanisms underlying the onset and development of MASLD/MASH.

**Figure 2:**
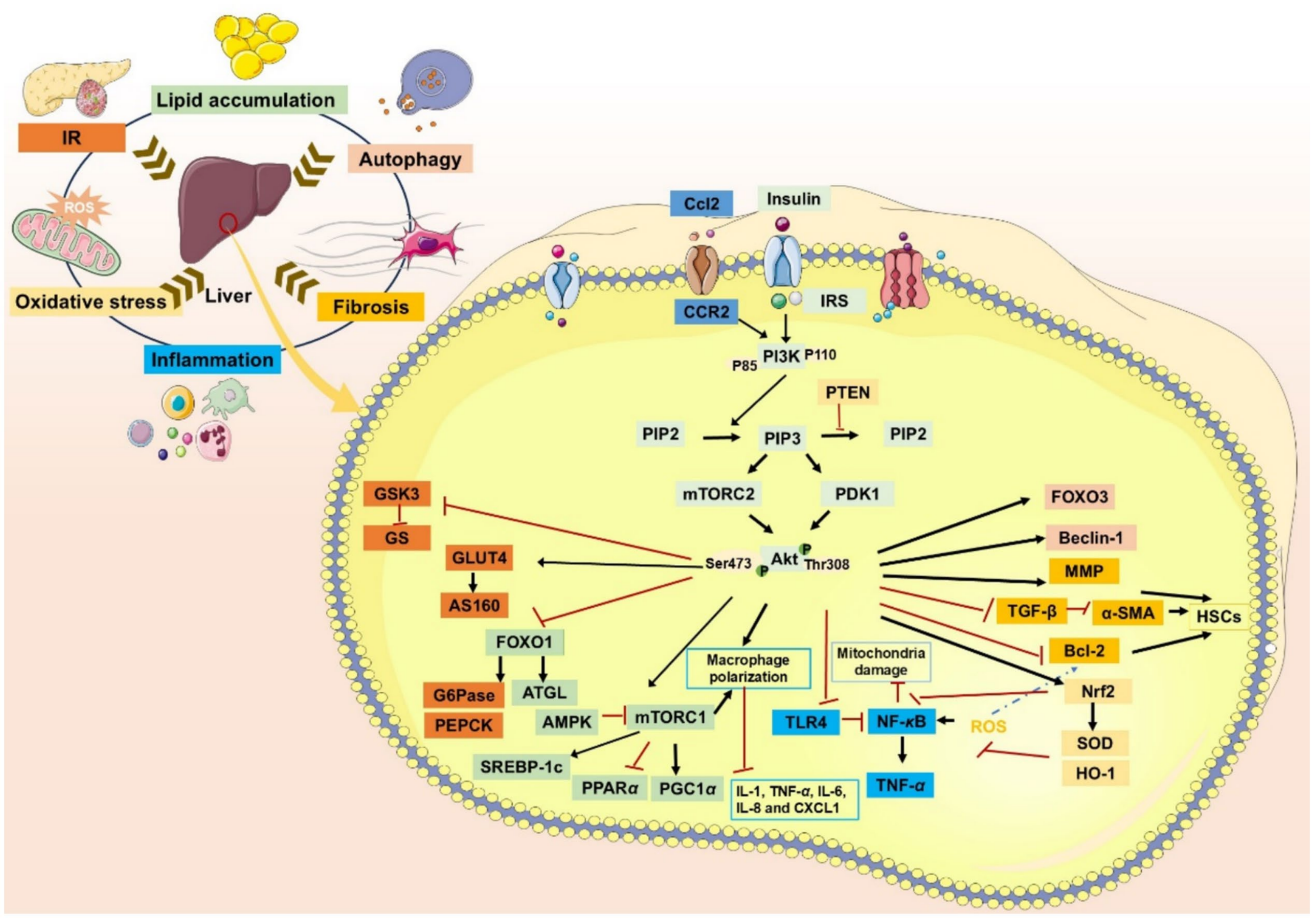
The role of PI3K/AKT signaling pathway in MASLD/MASH.

In the following sections, we will summarize the role of the PI3K/AKT signaling pathway in hepatic metabolism to elucidate the mechanisms underlying the development and progression of MASLD/MASH (Figures 1, 2).

### 2.1 Role of PI3K/AKT signaling pathway in lipid metabolism

The abnormal and excessive accumulation of lipid droplets within hepatocytes plays a fundamental role in the pathogenesis of MASLD and its progression to MASH (4). The lipid storage capacity of the liver is determined by multiple processes, including *de novo* lipogenesis (DNL), fatty acid uptake, triglyceride synthesis, fatty acid oxidation, and triglyceride export (22). Among them, increased lipid synthesis is a key feature of fatty liver. Study has reported that DNL in MASLD/MASH patients may be up-regulated by 20–30% compared with healthy controls (23). In addition, DNL is closely related to excessive glucose intake and the occurrence of IR, which further contributes to the development of MASLD/MASH. Essential for DNL are fatty acid synthase (FAS) and acetyl-CoA carboxylase (ACC), which are regulated by sterol regulatory element-binding protein 1c (SREBP-1c) (24). In the AKT-mediated lipid metabolism process, key regulatory substrates include SREBP and FOXO1. SREBP mainly regulates the expression of genes involved in FAS production and cholesterol homeostasis. On the contrary, FOXO1 facilitates lipolysis through transcriptional upregulation of adipose triglyceride lipase (25, 26).

AKT stimulates SREBP-1c activity through a cascade of mTORC1-dependent and mTORC1-independent signaling, while inhibiting AMP-activated protein kinase (AMPK) activity to regulate liver DNL. Recent evidence further indicates that mTORC1 activation, which is dependent on AKT, is essential for the induction of DNL (22). In MASLD/MASH, impaired adipose tissue responsiveness reduces free fatty acid (FFA) uptake and glucose utilization, resulting in ectopic lipid deposition in non-adipose organs. Meanwhile, the dysregulation of adipocyte cytokines and adipocyte hypertrophy hinders the PI3K/AKT signaling pathway, impairs SREBP activity, and reduces glucose metabolism, thereby exacerbating IR (19). mTORC1, as a key regulator of the metabolic adaptive switch, exhibits a tight and reciprocal regulation with AMPK and AKT, which can counteract MASLD accompanied by obesity.

There are relatively few studies on FOXO1 in liver lipid metabolism. Research has demonstrated that FOXO1 is directly implicated in the insulin-promoted expression of adipogenicity genes in the liver. Moreover, multiple studies have further substantiated its facilitating role in these metabolic processes (27–29). Nevertheless, emerging evidence indicated that the suppression of FOXO1 serves as both a necessary and sufficient prerequisite for the activation of DNL (30).

The PPAR gamma coactivator-1 alpha (PGC-1α)/PPARα axis is another crucial pathway for hepatic lipid metabolism that depends on the PI3K/AKT signaling pathway and is modulated by feeding and fasting cycles (22). In the liver, PPAR*α* plays an essential role in fatty acid catabolism. However, the expression of its target genes cannot be fully activated by PPAR*α* alone and requires the involvement of PGC-1*α* (31). The PGC - 1*α*/PPAR*α* complex modulates a multitude of genes implicated in fatty acid transport and β-oxidation, which are indispensable processes for sustaining fatty acid homeostasis during periods of fasting (32–34).

### 2.2 Role of PI3K/AKT signaling pathway in the IR

IR describes a pathophysiological state in which key metabolic organs exhibit a compromised reactivity to insulin signaling. IR is essential for activating the lipotoxic, oxidative stress, and inflammatory cascades that contribute to MASLD progression (35). Notably, IR originating in the liver subsequently promotes its development in peripheral tissues, including skeletal muscle and adipose tissue (36, 37).

Upon engagement with its receptor, insulin triggers the initiation of the insulin receptor substrate (IRS)/PI3K/AKT signal transduction cascade. This pathway serves as the fundamental mechanism through which insulin exerts its regulatory functions in hepatic physiology (38). Research has demonstrated that IRS2-knockout mice develop “selective IR,” whereas the absence of IRS1 or the simultaneous knockout of both IRS1 and IRS2 results in “total IR.” This result indicates that IRS1/2 are key factors in triggering selective IR (39). FFAs and liver lipid metabolites impair hepatic glucose metabolism by disrupting insulin action in the liver. This subsequently led to a dysfunction in insulin signaling, resulting in impaired glucose uptake, failure to activate glycogen synthesis, and failure to inhibit gluconeogenesis (40). GSK3, which is the earliest discovered substrate of AKT, consists of two isoforms known as GSK3α and GSK3β (41). GSK3*α* is critically involved in regulating glycogen synthesis, whereas GSK3β engages in a variety of biological processes such as the development, proliferation, and apoptosis of tumor cells (42, 43). Moreover, the inactivation of GSK3 can lead to the activation of glycogen synthase (GS) (44). Additionally, the PI3K/AKT signaling pathway may facilitate the conversion of glycogen phosphorylase (GP) from its active form (GPa) to its inactive state (GPb). Collectively, the activation of GS together with the inactivation of GP promotes an overall increase in glycogen synthesis (45). In addition, AKT directly phosphorylates AS160 (also known as TBC1D1), a 160 kDa substrate, to induce glucose transporter type-4 (GLUT4) transport from storage vesicles to the plasma membrane. Under insulin and AKT stimulation, glucose transport in skeletal muscle is then completed (46, 47).

Under IR conditions, the upstream transcription factor SREBP-1 becomes activated and enhances the expression of key lipogenic enzymes including FASN. This upregulation subsequently stimulates the production of lipids such as total triglyceride (TG), total cholesterol (TC), and FFA (48). Conversely, excessive accumulation of FFAs stimulates the production of pro-inflammatory factors, which in turn activate multiple kinases and ultimately inhibit insulin signal transduction (49).

Accumulating evidence suggests that FOXO proteins, a subfamily of forkhead transcription factors, play a pivotal role in mediating how insulin and growth factors regulate various physiological functions (50). The FOXO subfamily includes at least three members: FOXO1, FOXO3a, and FOXO4, each of which is susceptible to AKT-mediated phosphorylation and subsequent inactivation. The AKT kinase phosphorylates FOXO transcription factors at several residues, including Thr32, Ser253, and Ser315. This phosphorylation promotes the nuclear exclusion of FOXO and suppresses its ability to regulate transcription (51). A study shows that fasting hyperglycemia and hepatic IR are observed in transgenic mice with liver-specific expression of constitutively active FOXO1. This effect is closely related to the upregulation of glucose-6-phosphatase (G6Pase) gene expression (52). AKT suppresses both the nuclear translocation and transcriptional activity of FOXO1, thereby decreasing gluconeogenesis through the downregulation of key enzymes such as G6Pase and phosphoenolpyruvate carboxy kinase (PEPCK) (53).

### 2.3 Role of PI3K/AKT signaling pathway in the inflammation

MASLD involves a variety of immune cell-mediated inflammatory processes, particularly when it reaches the MASH stage, at which point inflammation becomes an integral part of disease progression (54). Liver inflammation in MASH can be triggered by both extrahepatic and intrahepatic factors. External contributors include dysfunctional adipose tissue and intestinal disturbances, while internal hepatic mechanisms involve lipotoxic injury, dysregulated innate immunity, impaired mitochondrial function, and ER stress. Collectively, these pathways interact to promote the progression of MASH (55).

In recent years, an increasing number of studies have begun to elucidate how the PI3K signaling pathway modulates inflammatory responses associated with MASLD/MASH. Recent studies indicate that class I PI3K, which is highly expressed in leukocytes, plays a critical regulatory role in mediating the recruitment and activation of innate immune cells in inflammatory sites (56). C-C motif chemokine ligand 2 (CCL2) is the first member of the human CC chemokine family to be purified and biologically characterized. Generally, it interacts with C-C motif chemokine receptor 2 (CCR2) to exert biological functions (57). CCR2 is characterized as a member of the G protein-coupled receptor family and has been demonstrated to modulate various downstream signaling cascades, notably including the PI3K/AKT signaling pathway (58). According to recent studies, both CCL2 and CCR2 exhibit markedly elevated expression levels in the hepatic tissues of MASH mouse models and MASLD patients, accompanied by a substantial increase in the infiltration of pro-inflammatory cells within the liver (59, 60). Wu et al. (61) have demonstrated that the CCL2/CCR2 axis regulates lipid metabolic dysregulation, release of inflammatory mediators, and hepatic inflammatory responses in MASH through activation of the downstream PI3K/AKT signaling pathway. Consistent with these results, a separate study also reveals that miR-122 knockout, which inhibits the CCL2-CCR2 axis, effectively attenuates inflammatory cell accumulation in the liver and reduces tumor necrosis factor-α (TNF-α) production. This effect is achieved by suppressing the nuclear transcription factor-*κ*B (NF-*κ*B) inflammatory signaling pathway (62).

The PI3K/AKT signaling pathway plays a critical role in regulating macrophage polarization. Under inflammatory conditions, activation of this pathway facilitates the transition of microglia from a pro-inflammatory M1 state to an anti-inflammatory M2 phenotype. These alterations lead to a marked upregulation of anti-inflammatory mediators, including interleukin-10 (IL-10), IL-1ra, and interferon-β, alongside a substantial downregulation of pro-inflammatory cytokines such as IL-1, TNF-*α*, IL-6, IL-8, and CXCL1 (63). Toll-like receptor 4 (TLR4), a transcriptional target of FOXO1, drives the polarization of macrophages and microglia toward the M1 phenotype. This polarization is accompanied by enhanced production of proinflammatory mediators, primarily through TLR4-dependent activation of the NF-*κ*B signaling pathway. Research indicates that the activation of the PI3K/AKT signaling pathway contributes to the downregulation of TLR4 expression, thereby suppressing the production of inflammatory mediators (64). mTOR also plays a critical role in regulating macrophage polarization. Pharmacological inhibition of mTOR using rapamycin is found to enhance the production of pro-inflammatory cytokines (including IL-1β, TNF-α, and IL-6) in M1 macrophages. Conversely, the same treatment promotes apoptotic activity in M2 macrophages (65). Besides, activated AKT induces mTOR activation by phosphorylating and inactivating the tuberous sclerosis complex (TSC). The subsequent inhibition of TSC2 is associated with three primary effects: suppressed NF-*κ*B expression, enhanced signal transducer and activator of transcription 3 (STAT3) activity, and a decline in pro-inflammatory cytokine production (66). It has been reported that activation of the PI3K/AKT pathway in hepatocytes typically reduces inflammatory responses by suppressing NF-*κ*B expression (67–70). Despite the findings presented here, the relationship between the PI3K/AKT signaling pathway and NF-*κ*B is controversial. Evidence from previous studies indicates that the activation of the PI3K/AKT signaling pathway upregulates NF-*κ*B-mediated transcription. Consequently, this molecular event drives the expression of pro-inflammatory genes in HeLa cells, ME - 180 cells, and macrophages (71, 72). These conflicting findings indicate that the PI3K/AKT signaling pathway activates NF-*κ*B through pleiotropic, cell-type-specific mechanisms, with the non-coordinate expression of I*κ*B kinases (IKKα and IKKβ) contributing to this cell-type-specific role of AKT (73). In short, the PI3K/AKT signaling pathway serves a critical function in modulating the inflammatory response associated with the progression of MASLD and MASH (74).

### 2.4 Role of PI3K/AKT signaling pathway in the oxidative stress

Of all pathophysiological mechanisms implicated in MASH, oxidative stress remains the most extensively investigated (75). Clinical analyses of MASH-affected liver samples reveal a significant correlation between elevated oxidative stress markers, increased neutrophil presence, and the extent of histopathological damage (76). In MASLD, excessively accumulated fatty acids are oxidized by microsomal enzymes, leading to the production of reactive oxygen species (ROS) (77). Then, increased production of ROS induces apoptosis and amplifies inflammation, thus inflicting the second hit on the liver (78). Evidence from earlier studies demonstrates that elevated ROS levels induce NF-*κ*B-mediated inflammation in liver cells. The subsequent upregulation of inflammatory cytokines and chemokines further promotes the activation of both Kupffer cells and hepatic stellate cells (HSCs). This activation represents a crucial step in the transition from simple fatty liver disease to MASH (55, 62).

Nrf2 is referred to as the “master regulator” of the antioxidant response (79). Phosphorylation at the serine 40 residue promotes the activation of Nrf2, thereby initiating an antioxidant response mediated by the Nrf2 signaling pathway. Extensive research has established that Nrf2 modulates the transcription of key antioxidant enzymes, including superoxide dismutase (SOD) and heme oxygenase-1(HO-1). This regulation conferred cytoprotective effects against oxidative damage in hepatocytes, as demonstrated in animal models of MASLD (80). Besides, Jin et al. (81) have reported that the elevation in Nrf2 expression reduces inflammation and NF-*κ*B activity. In mammalian cells, the PI3K/AKT signaling pathway serves as a critical upstream regulator of Nrf2. Activation of PI3K/AKT facilitates the translocation of Nrf2 from the cytoplasm to the nucleus (3). Li et al. (82) have demonstrated that treatment with the PI3K inhibitor LY294002 markedly attenuates both the nuclear translocation of Nrf2 and the upregulation of HO-1 induced by S-propargyl-cysteine.

PTEN functions as a key negative regulator of the PI3K/AKT signaling pathway by dephosphorylating PIP3, thereby inhibiting AKT activation and downstream signaling processes (83). Researchers conclude that PTEN plays a significant role in insulin-induced oxidative stress and genomic instability. Experimental inhibition of PTEN has been shown to elevate intracellular ROS levels and exacerbate DNA damage *in vitro*. Similar results are also observed *in vivo*. In the mouse model, knockdown of PTEN results in increased levels of oxidative stress, especially in high-fat diet (HFD)-induced mice. This conclusion is supported by experimental measurements of ROS levels, expression of the stress-responsive proteins heat shock protein 70 (HSP70) and HO-1, and assessment of genomic integrity in hepatic tissue (84).

### 2.5 Role of PI3K/AKT signaling pathway in the autophagy

Autophagy, a lysosome-dependent cellular catabolic pathway, is essential for maintaining metabolic homeostasis in hepatocytes. Impairment of hepatic autophagy and lysosomal function has been implicated in the development of pathogenic steatosis associated with MASLD (85). Research has demonstrated that both genetic and diet-induced mouse models of obesity exhibit impairments in hepatic autophagy and chaperone-mediated autophagy. These dysregulated autophagic processes are further implicated in the development of obesity-associated hepatic steatosis and IR (86–88). Importantly, emerging evidence indicates that autophagic flux and lysosomal activity are compromised in the livers of individuals diagnosed with MASLD (89, 90).

There are many pathways involved in autophagy. mTOR is a key regulator of growth and metabolism, influencing gene expression *via* direct interactions with transcription factors, epigenetic enzymes, and chromatin remodelers (91, 92). Moreover, the mTOR signaling pathway serves as a central regulatory checkpoint that negatively controls autophagy. Dysregulation of the mTOR pathway disrupts autophagic activity, resulting in mitochondrial dysfunction and diminished glucose uptake, which collectively contribute to the progression of MASLD (93, 94). And the PI3K/AKT/ mTOR pathway, as a critical regulator of autophagy, has been demonstrated to regulate the autophagy process. Generally, activation of class I PI3K signaling suppresses autophagy through the well-established PI3K/AKT-mTORC1 pathway. The catalytic subunit of class III PI3Ks, PIK3C3/Vps34, associates with BECN1 and PIK3R4 to form a multiprotein complex. This complex is responsible for the production of phosphatidylinositol 3 - phosphate, a lipid second messenger that plays an indispensable role in both the initiation and advancement of autophagy. The class II PI3K enzyme has recently been identified as an alternative source of phosphatidylinositol 3-phosphate and a novel regulator of autophagy initiation (95, 96). Sun et al. (97) demonstrate that pharmacological inhibition of the PI3K/AKT/mTOR signaling pathway induces autophagic activation, which subsequently attenuates intracellular lipid accumulation, suppresses inflammatory responses, and ameliorates MASLD.

Oxidative stress is one of the main causes of autophagy in cells. Under conditions of moderate oxidative stress, both mTORC1 and mTORC2 complexes function to suppress autophagic activity. However, under severe oxidative stress, mTORC2 can promote cellular senescence through autophagy-dependent mechanisms (98). Shen et al. (99) demonstrate that follicle-stimulating hormone has the capacity to suppress oxidative stress-induced autophagy. This inhibitory effect is primarily mediated through the activation of the PI3K/AKT/mTOR signaling pathway. Conversely, subsequent research indicates that pharmacological inhibition of mTOR, using specific blockers, results in the induction of autophagy (100). Besides, the effect of PTEN may vary depending on whether it is continuously silenced under basal conditions or transiently upregulated under acute oxidative stress. The elevated levels of ROS initiate a feedback mechanism that promotes a transient upregulation of PTEN. AKT directly phosphorylates beclin-1 at the Ser295 residue, resulting in its functional inactivation. The upregulation of PTEN inhibits this site-specific phosphorylation, thereby preserving beclin-1 activity (101).

FOXO transcription factors modulate autophagy through multiple mechanisms, such as binding to autophagy-related gene promoters (102), directly interacting with cytoplasmic autophagy proteins (103), and participating in epigenetic regulation (104, 105). FOXO1 and FOXO3, which are connected in autophagy induction, are members of the FOXO transcription protein family with autophagy-regulating effects. FOXO3 significantly upregulates the expression of PIK3CA, the catalytic subunit of class I PI3K, thereby enhancing AKT1 activation. Subsequently, activated AKT1 catalyzes the phosphorylation of FOXO1, leading to its translocation into the cytoplasm and triggering autophagy (106). Zhang et al. (107) demonstrate that Punicalagin enhances the expression of key autophagy-related proteins, including LC3b and p62, and mitigates T2DM-induced liver injury by reactivating autophagy *via* the AKT/FOXO3a signaling axis.

### 2.6 Role of PI3K/AKT signaling pathway in the fibrosis

Fibrosis, which is pathologically defined by the excessive deposition of extracellular matrix (ECM) components such as collagen, constitutes a critical pathological mechanism in the progression of MASLD. Accumulating evidence has established that HSCs serve as the principal producers of ECM, and their activation represents a central event in the development of liver fibrosis (108). If fibrosis continues unopposed, it disrupts the normal architecture of the liver and alters its normal functioning (109). Recently, studies have suggested that the fibrosis stage can more reliably predict liver-specific mortality compared to the MASLD activity score (110, 111). In the context of liver fibrosis attenuation, the PI3K/AKT signaling pathway has been identified as a critically involved mechanism, revealing promising directions for therapeutic intervention.

Inhibition of HSC activation is regarded as a key therapeutic strategy for reversing liver fibrosis (108). Current evidence underscores the significance of PI3K/AKT/mTOR-regulated autophagy in the reversal of liver fibrosis. Numerous studies have demonstrated that enhanced autophagy in HSCs promotes their activation. This occurs through the degradation of lipid droplets, a process that supplies energy to fuel HSC activation (87, 112, 113). For instance, Huang et al. (114) report that insulin-like growth factor-binding protein-related protein 1 (IGFBPrP1) facilitates the activation of HSCs by enhancing autophagy through the PI3K/AKT/mTOR signaling pathway. However, findings from Zhang et al. (115) reveal that enhanced autophagic flux in HSCs suppresses their activation. Similarly, Lee et al. (116) have found that stimulating autophagy *via* the PI3K/AKT/mTOR pathway inhibits the activation of non-chemically induced hepatic stellate cells (NHSCs). The underlying reasons for these discrepant findings remain unclear. This apparent discrepancy may be attributed to varying levels of autophagic activity in HSCs. Different autophagy inducers (e.g., IGFBPrP1, TGF-β, methyl helicterate) are employed in the studies mentioned above, which may have led to varying degrees of induction effect. Moderate autophagy supports HSC activation by supplying energy, whereas excessive autophagy induces autophagic cell death, thereby inhibiting their activation (117). Additionally, it is recommended that future research use the expression levels of *α*—SMA and type I collagen as the gold standards for HSC activation assessment to standardize evaluation metrics.

FOXO1, which can be regulated by the PI3K/AKT signaling pathway, also plays a crucial role in the trans-differentiation and proliferation of HSCs in liver fibrosis (111). Studies have found that FOXO1 inactivation caused by hyperinsulinemia promotes the proliferation and trans-differentiation of HSCs. Consistent with the above conclusion, FOXO1+/− mice have increased susceptibility to experimental liver fibrosis (118). It is noteworthy that FOXO1 inactivation exerts opposing effects in HSCs compared to hepatocytes. While FOXO1 activation in hepatocytes drives gluconeogenesis and subsequent IR, its expression and functional role are reconfigured in activated HSCs. These findings reveal the cell-type specificity of FOXO1 function. Besides, FoxO1 activity is context-dependent, as evidenced by its distinct functions in immature versus mature B cells (119). Moreover, apoptosis, or programmed cell death, plays an essential role in the resolution of liver fibrosis. Shifts in the balance between a subset of pro-apoptotic and pro-survival proteins, such as B-cell lymphoma 2 (BCL-2), which undergoes phosphorylation upon AKT activation, can initiate the apoptotic cascade. This cascade subsequently leads to the elimination of activated HSCs and the consequent amelioration of fibrosis (14, 120, 121).

The activation of AKT signaling inhibits the expression of key profibrotic genes, such as transforming growth factor beta (TGF-β) and alpha-smooth muscle actin (α-SMA), in HSCs. Additionally, it suppresses the activity of NF-*κ*B, a transcription factor that plays a pivotal role in inflammation and fibrosis processes (122). In addition to the inflammatory response, oxidative stress also significantly affects the progression of liver fibrosis. The activation of the PI3K/AKT signaling pathway can promote the expression of antioxidant enzymes, which is conducive to scavenging reactive oxygen species and resisting oxidative damage (123). In addition, liver fibrosis is associated with inadequate degradation of ECM proteins, which can be remodeled by the PI3K/AKT signaling pathway. Research indicates that AKT activation upregulates the synthesis and function of matrix metalloproteinases (MMPs), thereby facilitating the degradation of ECM proteins (124).

## 3 Targeting the PI3K/AKT signaling pathway for MASLD/MASH therapy

Accumulating research has explored the impact of herbal medicines on MASLD/MASH. Evidence indicates that bioactive constituents isolated from herbal medicines exhibit considerable potential in mitigating MASLD/MASH by modulation of the PI3K/AKT signaling pathway. The following section details the mechanisms by which the PI3K/AKT signaling pathway participates in protecting against MASLD/MASH, with a particular focus on the actions of herbal medicines and their isolated monomers.

### 3.1 Monomers from herbal medicines

#### 3.1.1 Phenylpropanoids

The phenylpropanoids, a class of secondary metabolite compounds characterized by a phenyl group, are natural products found in many aromatic and medicinal plants, foods, and essential oils. Due to the extensive pharmacological effects, including anti-tumor, anti-inflammatory, antibacterial, and antioxidant effects, phenylpropanoids hold enormous potential for application within the pharmaceutical industry (125). Curcumin, a known natural compound from turmeric, has been demonstrated to reduce oxidative stress and inflammation in obesity, T2DM, and MASLD. Dihydrocurcumin and tetrahydrocurcumin are the major metabolites of curcumin. In 2023, research by Wu et al. (126) indicated that the hepatoprotective effects of curcumin, including the amelioration of lipid accumulation, inflammation, and endothelial dysfunction, were mediated by the inhibition of the NF-*κ*B and PI3K/AKT/ hypoxia inducible factor-1 alpha (HIF-1α) signaling cascades. This study further revealed that antibiotics diminished these benefits by reducing the biosynthesis of tetrahydrocurcumin, a key intestinal metabolite of curcumin. Importantly, tetrahydrocurcumin exhibited superior efficacy to curcumin in restoring liver sinusoidal endothelial cell function and attenuating steatosis and cellular injury in human normal hepatocytes (L02) models (126). As first reported by Holder et al. (127) tetrahydrocurcumin is a principal metabolite formed by the reductive metabolism of curcumin. Subsequent studies on this compound demonstrate that tetrahydrocurcumin pretreatment also significantly suppresses hepatic steatosis in hepatocellular carcinoma (HepG2) cell cultures. Furthermore, tetrahydrocurcumin treatment restores glucose uptake and insulin signaling, which have been impaired in oleic acid (OA)-incubated HepG2 cells. This effect includes phosphorylation of the IRS-1/PI3K/AKT pathway and downstream signaling pathways (such as FOXO1 and GSK3β), which are involved in gluconeogenesis and glycogen synthesis (128). In addition, Yu et al. (129) reported that dihydrocurcumin, another metabolite of curcumin, ameliorated OA-induced steatosis through the regulation of lipid metabolism, oxidative stress, and IR in HepG2 and L02 cells. The therapeutic impact of dihydrocurcumin could be ascribed to its influence on critical regulatory pathways, encompassing genes for lipid metabolism [PPAR*α*, SREBP-1C, and patatin-like phospholipase domain-containing 3 (PNPLA3)], the Nrf2-mediated oxidative stress response, and PI3K/AKT-dependent insulin signal transduction. Collectively, the present findings indicate that the mitigation of MASLD/MASH by curcumin and its metabolic derivatives is mediated by the PI3K/AKT signaling pathway. However, despite the encouraging results from preclinical studies, the translational potential of curcumin and its metabolic derivatives in clinical practice remains to be fully validated. Furthermore, individual variations in gut microbiota composition may affect the generation of curcumin metabolites, which ultimately results in significant variations in their plasma levels among humans. This limitation underscores the need for rigorous, large-scale, placebo-controlled clinical trials using standardized and optimized curcumin formulations to truly assess its efficacy and safety in humans.

Scoparone is a naturally bioactive coumarin isolated from the Chinese herb *Artemisia capillaris*. It has been shown to ameliorate various forms of liver disease. Liu et al. (130) demonstrated that scoparone enhanced autophagic function and ameliorated multiple core pathological manifestations of MASH in mice subjected to a methionine-choline-deficient (MCD) diet. *In vitro* analyses revealed that scoparone influenced autophagy in macrophages, yet it exhibited no significant effect on hepatocyte autophagic processes. Inhibition of the PI3K/AKT/mTOR pathway, coupled with an increase in autophagic flux, constituted a probable mechanism through which scoparone exerted its protective effects on MASH (130). Research has indicated that esculetin, a dihydroxy coumarin derivative, exerts pronounced anti-fibrotic effects in the livers of MASLD model rats. This activity is potentially mediated through the activation of the PI3K/AKT/FOXO1 pathway, evidenced by a marked downregulation in phosphorylated FOXO1 expression (111).

Arctigenin, a lignan derived from *Arctium lappa* (L.), possesses both antioxidant and anti-inflammatory properties. Chen et al. (33) noted that arctigenin suppressed the progression of MASLD *in vitro* by attenuating lipotoxicity and inflammation induced by lipid oxidation. Further mechanistic studies found that the effect of arctigenin in reducing intracellular oxidative stress was mediated by the PI3K/AKT pathway. Daphnetin is a compound extracted from the medicinal plant *Daphne koreana* Nakai. In OA-induced HepG2 cells, the compound was shown to reduce lipid accumulation, IR, and oxidative stress, thereby supporting its potential for ameliorating multiple symptoms of MASLD pathology. Mechanistically, activation of the PI3K/AKT pathway was a key mechanism through which daphnetin mitigated IR (131).

#### 3.1.2 Flavonoids

Defined by a C6-C3-C6 structural backbone, flavonoids are hydroxylated phenolic compounds that serve as secondary metabolites in plants. They are extensively dispersed across diverse flora, and more than 4,000 naturally occurring flavonoids have been documented in various plant species and berries (132, 133). Among natural products, flavonoids have been extensively studied. They demonstrate significant potential as one of the most promising and important agents to treat cancer, oxidative stress, infections caused by pathogenic bacteria, inflammations, cardiovascular dysfunctions, and other conditions (134). More recently, flavonoids have shown potential for use in therapeutic and preventive strategies against MASLD/MASH.

Flavone, a highly representative flavonoid, exhibits an extensive spectrum of bioactivities. Based on network pharmacology and subsequent experimental validation, Chen et al. (135) demonstrated that luteolin, a key bioactive metabolite derived from the *Salvia miltiorrhiza Bunge*-*Reynoutria japonica Houtt.* herb pair (SRDP), significantly attenuated lipid accumulation and reduced intracellular triglyceride levels in a HepG2 cell model of MASLD. The study revealed that luteolin suppressed the mTOR signaling pathway by inhibition of phosphorylation within the PI3K/AKT axis, which in turn promoted autophagy activation and ameliorated MASLD. Interestingly, a study found that the nano-formulation of luteolin with Zn oxide as Lut/ZnO NPs might improve the anti-MASLD properties of each component alone. The administration of Lut/ZnO NPs resulted in the activation of the PI3K/AKT/FOXO1 signaling pathway. This activation promoted FOXO1 phosphorylation, thereby suppressing its translocation into the nucleus and impairing its transcriptional activity. As a consequence, hepatic gluconeogenesis was reduced through the downregulation of G6Pase gene expression, ultimately leading to an amelioration of IR in rats with MASLD (136). Scutellarin, the primary bioactive constituent of breviscapine, was investigated by Zhang *et al.* for its protective potential using an MASLD model induced by an HFD in combination with chronic stress. The results indicated that scutellarin significantly ameliorated hepatic lipid metabolism and attenuated oxidative stress damage. Its antioxidant effects against MASLD were likely mediated through activation of the PI3K/AKT pathway with subsequent Nrf2 nuclear translocation, which increased expression of HO-1 and NAD(P)H quinone oxidoreductase 1 (NQO1) (137).

Isoflavones represent a widespread subclass of flavonoids commonly found in species belonging to the *Leguminosae* family. Puerarin is a major bioactive isoflavone compound isolated from the roots of the *Pueraria lobata* and has been clinically used for its protection against inflammation, oxidative stress, and mitochondrial dysfunction. Wang et al. (117) demonstrated that puerarin downregulated poly (ADP-ribose) polymerase 1 (PARP-1) expression in a mouse model of MASLD induced by the high-fat and high-sugar diet (HFHSD). The protective effects of puerarin were replicated by PJ34, a specific PARP inhibitor. However, pharmacological inhibition of PI3K abolished the ability of both puerarin and PJ34 to restore NAD + levels and maintain mitochondrial function, thereby exacerbating hepatic steatosis and metabolic dysregulation induced by HFHSD (117). In a separate investigation, Yang et al. (138) observed that hepatic ischemia–reperfusion injury (IRI) was exacerbated in fatty liver tissue compared to normal liver. Puerarin administration markedly attenuated IRI by reducing apoptosis and suppressing ROS production in a MASLD mouse model. Mechanistic investigations revealed that puerarin conferred substantial protection against hepatic IRI in MASLD models through reactivation of the PI3K/AKT signaling pathway. Moreover, the protective effects of puerarin were significantly diminished by LY294002, a specific inhibitor of PI3K/AKT (138). Recently, Fang et al. (139) identified puerarin as a promising therapeutic agent for MASH treatment. Mechanistic analysis revealed that puerarin promoted the polarization of macrophages from the pro-inflammatory M1 phenotype to the anti-inflammatory M2 phenotype through activation of autophagic flux. Simultaneously, the PI3K/AKT signaling pathway was activated, promoting an increase in M2 macrophages. This modulation reduced pro-inflammatory factors and increased anti-inflammatory factors to exert the anti-MASH pharmacological effect (139). Collectively, these findings establish puerarin as a promising therapeutic candidate for the treatment of MASLD/MASH.

Liquiritigenin (LQ), a dihydrochalcone-type flavonoid derived from licorice root, has been widely reported to exhibit hepatoprotective properties. For example, studies have demonstrated that LQ ameliorates lipid accumulation, improves IR, and attenuates inflammatory responses in mouse models of MASLD. These beneficial effects are potentially mediated through activation of the PI3K/AKT signaling pathway (140). Farrerol, a natural dihydrochalcone flavonoid known for its antioxidant and anti-inflammatory activities, has recently attracted considerable research interest owing to its potential hepatoprotective effects. In the context of insulin signal transduction, protein tyrosine phosphatase non-receptor type 1 (PTPN1) acts as a key negative regulator. This cytoplasmic protein tyrosine phosphatase inhibits insulin receptor activity by dephosphorylating its tyrosine residues, thereby attenuating the transmission of the downstream PI3K/AKT signaling pathway. Gao et al. (141) reported that farrerol improved IR and alleviated lipid accumulation by binding to PTPN1 and reducing the dephosphorylation of the insulin receptor in HepG2 cells and MASLD mice. Thus, the PI3K/AKT signaling pathway was activated, leading to a reduction in downstream proteins involved in lipid synthesis (141). Notably, this protective effect was abolished with PTPN1 overexpression. In 2020, a study demonstrated that didymin markedly alleviated hepatic steatosis and hepatocyte injury induced by dexamethasone /HFD, primarily through suppression of the TLR4/NF-*κ*B and PI3K/AKT signaling pathways. These findings suggested the potential of didymin as a therapeutic agent for MASLD (142). Hesperetin, another dihydrochalcone flavonoid, serves as a key bioactive compound in aged citrus peel (known as chenpi in traditional Chinese medicine). This herbal material has been employed in the treatment of disorders related to glucose and lipid metabolism, including obesity, dyslipidemia, and cardiovascular conditions. Li et al. (68) demonstrated that hesperetin effectively attenuated hepatic oxidative stress both *in vivo* and *in vitro* by activating the PI3K/AKT-Nrf2 pathway. This antioxidant activity subsequently led to the suppression of NF-*κ*B-mediated inflammation, thereby mitigating the progression of MASLD.

Isoliquiritigenin, a chalcone-type flavonoid isolated from licorice root, serves as an isomeric precursor to liquiritigenin. Owing to its broad spectrum of biological and pharmacological activities, isoliquiritigenin has become one of the most extensively investigated compounds originating from Chinese herbal medicine. This compound has been shown to reduce hepatic lipid accumulation, inflammation, and fibrogenesis in a diet-induced mouse model of MASH. Moreover, isoliquiritigenin exerts protective effects against MASH and liver fibrosis by inducing autophagy through modulation of the PI3K/AKT/mTOR pathway. This finding suggests its potential as a therapeutic candidate for treating MASH and related fibrotic diseases in humans (143).

Flavanols, a distinct class of flavonoids ubiquitously present in numerous plants, exhibit a wide range of biological activities. Previous research has indicated the potential role of green tea in obesity prevention, with epigallocatechin gallate (EGCG) identified as one of its primary bioactive constituents. EGCG can prevent MASLD by alleviating liver inflammation in HFD-induced rats and by improving insulin signaling. Moreover, EGCG also increases the expression levels of PI3K, AKT, IRS-1, and IRS-2, all of which are vital in insulin signaling (144).

Kaempferol is one of the most common dietary flavonols. In 2022, Zhou et al. demonstrated that kaempferol modulated lipid metabolism, inhibited apoptosis, and regulated inflammatory responses and autophagy in a fatty liver cell model through activation of the PI3K/AKT signaling pathway (145).

#### 3.1.3 Terpenoids

Terpenoids, a class of naturally occurring compounds with substantial medicinal potential, are extensively distributed across diverse plant species. Ginseng, a highly valued medicinal herb, has been widely utilized in traditional Asian medicine for the treatment of various ailments. *Panax ginseng* saponins, recognized as the primary bioactive constituents of ginseng, play a significant role in modulating antioxidant, anti-inflammatory, and anti-tumor pathways. Ginsenoside Re, a distinctive tetracyclic triterpenoid present in ginseng, has been reported to ameliorate MASLD by restoring lipid homeostasis. In a recent study, Zhang et al. (146) employed integrated bioinformatics and experimental validation to elucidate the underlying mechanisms of ginsenoside Re in the treatment of MASLD. The results showed ginsenoside Re suppressed the expression of key proteins and mRNAs involved in PI3K/AKT-regulated lipogenesis and the TLR4/NF-*κ*B-driven inflammatory pathway, specifically PPAR*γ*, SREBP1c, ACC, FASN, and TLR4 (146). Ginsenoside Rk3, a recognized triterpenoid saponin, is among the bioactive ginsenosides derived from *Panax ginseng*. In a 2023 study, ginsenoside Rk3 was shown to markedly alleviate hepatic inflammation, lipid accumulation, and fibrotic changes in mice subjected to a high-fat-high-cholesterol (HFHCD) and carbon tetrachloride (CCl4) injections. Specifically, Guo et al. (147) demonstrated that ginsenoside Rk3 attenuated HFHCD-induced liver injury through modulation of the PI3K/AKT signaling pathway in the context of MASH. Taken together, the accumulated data provide strong evidence for ginsenoside Rk3 as a potential drug for treating MASLD/MASH.

As a naturally occurring pentacyclic triterpenoid saponin, Calenduloside E is a major saponin constituent extracted from the bark and root of *Aralia elata* (Miq.) Seem. Le et al. (148) revealed that calenduloside E administration significantly improved liver injury, lipid accumulation, inflammation, and pro-fibrotic markers in MASLD mice. Furthermore, transcriptomic profiling *via* RNA-seq revealed that the principal mechanism by which calenduloside E mitigated MASLD was mediated through inflammatory cascades associated with pyroptosis. And it was demonstrated that calenduloside E significantly attenuated inflammasome-induced pyroptosis in both cellular and animal models. Further experimental evidence confirmed that this compound exerted its inhibitory effect by suppressing the PI3K/AKT/NF-*κ*B signaling pathway (148).

In addition to triterpenes, certain diterpenes have also been shown to contribute to the treatment of MASLD/MASH through the PI3K/ AKT signaling pathway. Song et al. (149) found that carnosic acid, a phenolic diterpene, possessed the ability to alleviate HFD-induced MASLD in mice by reducing lipogenesis and inflammation in the liver. Myristoylated alanine-rich C-kinase substrate (MARCKS) represents a critically important substrate in varied cell types. Deficiency of MARCKS accelerates the progression of MASLD and further stimulate PI3K and phosphorylated AKT. Notably, carnosic acid (CA) treatment induces an upregulation of MARCKS expression in contrast to the HF group. This finding supports the potential efficacy of CA as a treatment for MASLD (149).

#### 3.1.4 Alkaloids

Alkaloids, which are secondary metabolites, are primarily isolated from plant sources and to a lesser extent from fungi and animals. Alkaloids derived from plants have begun to gain popularity worldwide for promoting health care as well as disease prevention (150).

Research has reported that sulforaphane present in broccoli florets, sprouts, and seeds, exhibits therapeutic potential against various metabolic diseases. In a 2022 study, findings from experimental research demonstrated that sulforaphane effectively reduced hepatic steatosis and mitigated liver injury in rats subjected to long-term HFD feeding. These findings were consistent with previous studies conducted in models of MASLD. And the novelty of the research was that the observed hepatoprotection was achieved, at least in part, *via* the activation of the PI3K/AKT signaling pathway (15). Lycorine, a natural alkaloid found in the bulbs of Lycoris species, exhibits significant anti-inflammatory, antiviral, and antitumor properties. However, its potential impacts on MASLD remain poorly characterized. Wang et al. (151) reported that lycorine administration resulted in a dose-dependent improvement in several MASLD-related indicators. Subsequent mechanistic investigation revealed that lycorine alleviated hepatic steatosis, oxidative stress, and ferroptosis through suppression of phosphorylated epidermal growth factor receptor (EGFR) expression, leading to inhibition of the PI3K/AKT signaling pathway. Lycorine was shown to stimulate the browning of white adipose tissue, thereby enhancing thermogenesis and increasing energy expenditure. Furthermore, lycorine administration was found to modulate gut microbiota composition, enhance intestinal barrier integrity, and attenuate intestinal inflammation. This study provides a new possibility for the treatment of MASLD (151).

#### 3.1.5 Others

**Figure 3:**
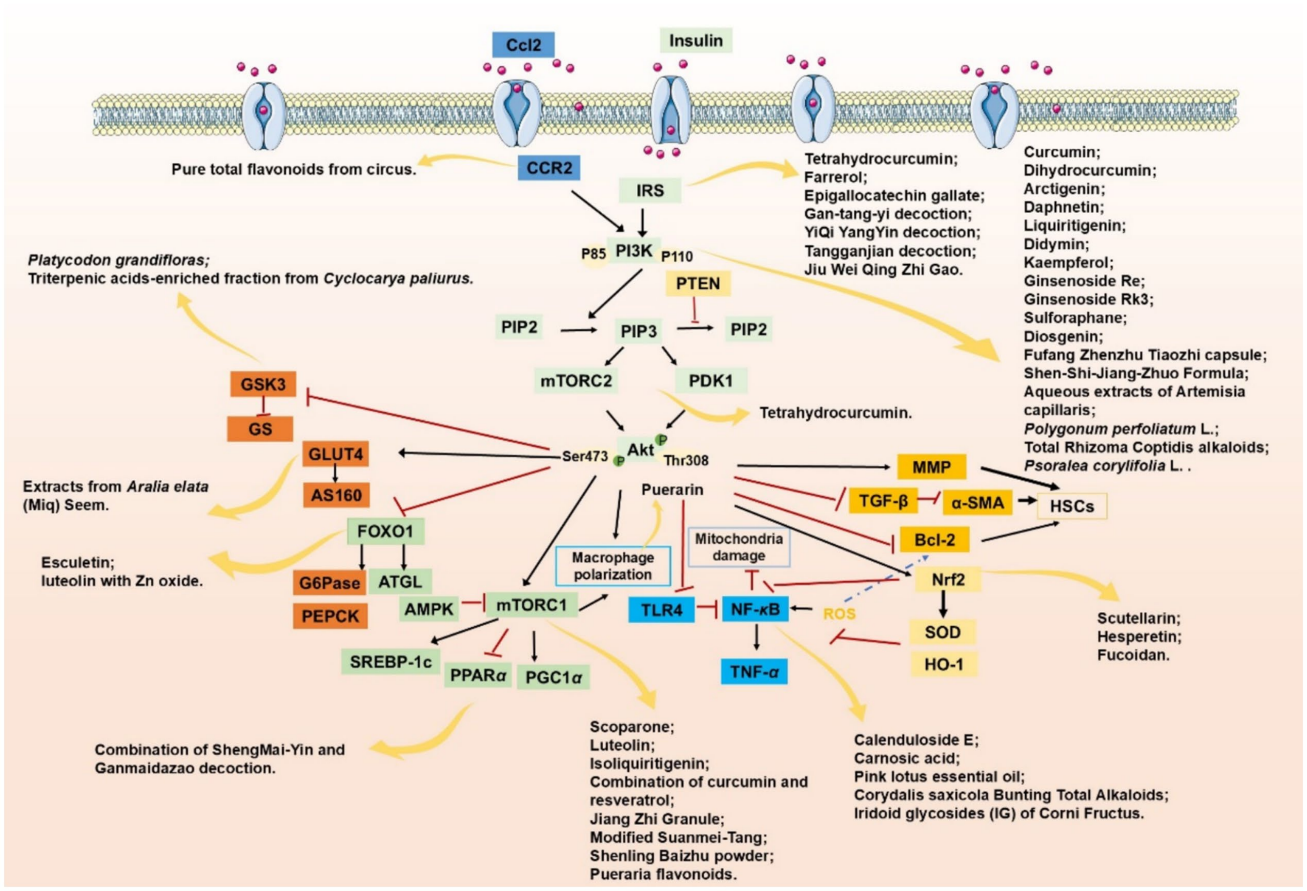
Modulation of the PI3K/AKT signaling pathway targeting MASLD/MASH through natural compounds.

In addition to the compounds mentioned above, there are many other natural agents with potential protective effects on MASLD/MASH *via* the PI3K/AKT signaling pathway. Diosgenin, a plant-derived steroidal sapogenin, has been suggested to promote fatty acid metabolism through modulation of the PI3K/AKT signaling pathway. *In vitro* evidence indicates that diosgenin treatment induced PI3K/AKT activation, downregulated stearoyl-CoA desaturase-1 (SCD1) expression, and led to reduced intracellular triglyceride accumulation and IL-6 secretion (152). In both spheroid and HepG2 cell models of MASLD, fucoidan alleviates FFA-induced lipid deposition, oxidative stress, and NF-*κ*B-driven inflammatory responses *via* activation of the PI3K/AKT/Nrf2 signaling pathway (67). Emerging evidence indicates that interactions between bioactive phytochemicals may produce advantageous synergistic effects. The combination of curcumin and resveratrol exhibits greater effects than those of curcumin and resveratrol used alone in the treatment of MASLD. Moreover, the underlying mechanism is found to be attributable, at least in part, to the regulation of the PI3K/AKT/mTOR and HIF-1 signaling pathways (153) (Table 1, Figure 3).

**Table 1:** Protective effects of monomers from herbal medicines on MASLD/MASH related to PI3K/AKT signaling pathway.

Agent	Chemical structure	Mechanism	In vitro activity/dose	In vivo activity/dose	Effects mediated by the PI3K/AKT signaling pathway	MASLD/MASH	Ref.
Curcumin	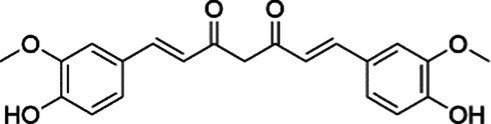	Inhibiting the PI3K/ AKT/HIF-1*α* signaling pathway	FFA-induced human liver sinusoidal endothelial cells (LSECs), 1, 2, 4, 8, and 10 μM	HFD-induced Sprague Dawley (SD) rats, 25, 50, 100 mg/kg	Ameliorating the chronic inflammation-induced endothelial dysfunction	MASH	(126)
Tetrahydrocurcumin	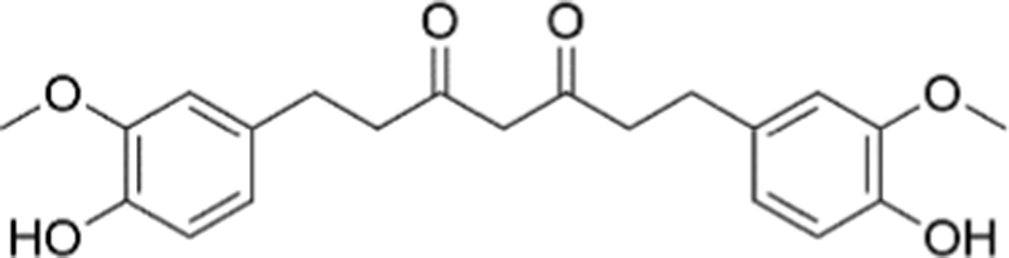	Activating the PI3K/AKT signaling pathway	OA induced HepG2 cells, 50, 100 μM	Not assessed	Alleviating IR	MASLD	(40)
Dihydrocurcumin	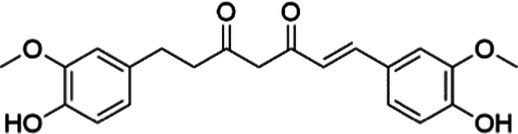	Activating the PI3K/AKT signaling pathway	OA-induced L02 and HepG2 cells, 5, 10, 20, 50 μM	Not assessed	Alleviating IR	MASLD	(129)
Scoparone	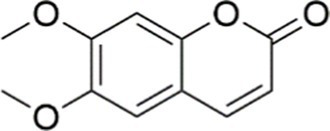	Inhibiting the PI3K/AKT/mTOR signaling pathway	Palmitic acid (PA)-induced AML12 cells, LPS-induced RAW 264.7 cells, 200 mM	MCD-induced C57BL/6 J mice, 20, 40, 80 mg/kg	Promoting autophagy	MASH	(130)
Esculetin	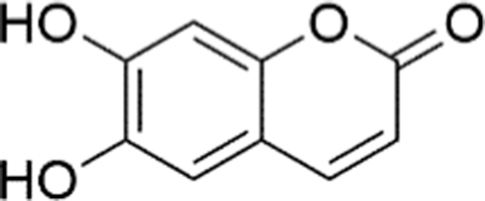	Activating AKT/PI3K/FOXO1 signaling pathway	Not assessed	HFD-induced Wistar rats, 50, 100 mg/kg	Reducing fibrosis	MASLD	(111)
Arctigenin	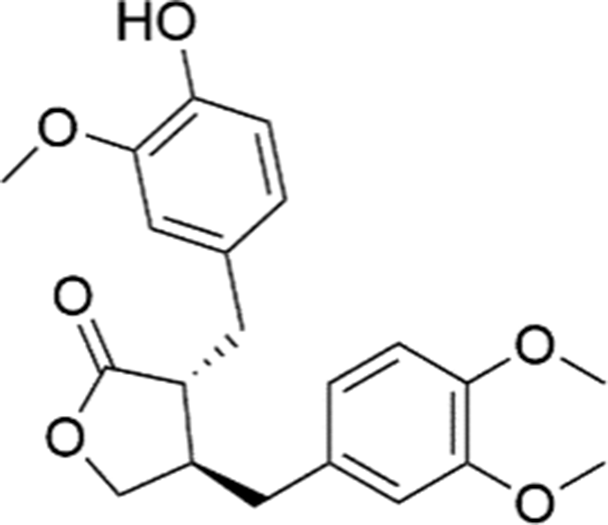	Activating the PI3K/AKT signaling pathway	OA-induced WRL68 hepatocytes, 50 μM	Not assessed	Reducing intracellular oxidative stress	MASLD	(128)
Daphnetin	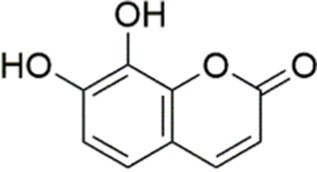	Activating the PI3K/AKT signaling pathway	OA-induced HepG2 cells, 5, 20, 50 μM	Not assessed	Alleviating IR	MASLD	(131)
Luteolin	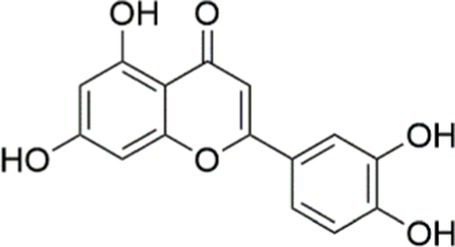	Inhibiting the PI3K/AKT /mTOR signaling pathway	PA-induced HepG2 cells, 20 mM	Not assessed	Activating autophagy	MASLD	(135)
Luteolin/ZnO NPs	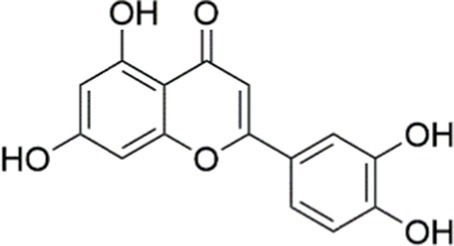	Activating the PI3K/AKT signaling pathway	Not assessed	HFD and streptozotocin-induced Wistar rats, 12 mg/kg	Alleviating IR	MASLD	(136)
Scutellarin	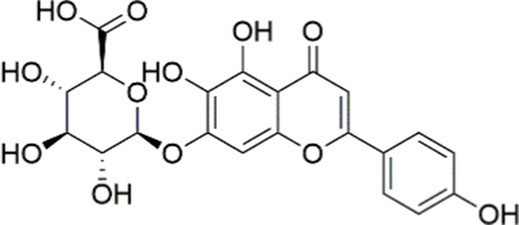	Activating the PI3K/AKT /Nrf2 signaling pathway	Not assessed	HFD-induced SD rats, 100, 300 mg/kg/d	Attenuating oxidative stress	MASLD	(137)
Puerarin	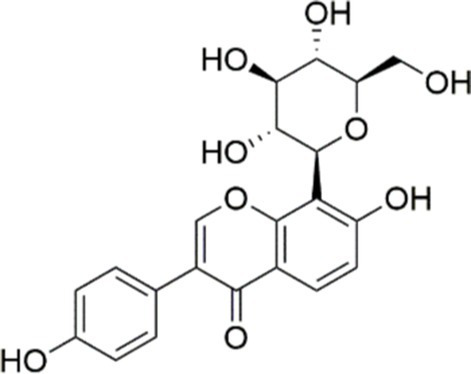	Activating the PI3K/AKT signaling pathway	Not assessed	HFHSD-induced C57BL/6 J mice, 0.2, 0.4 g/kg	Facilitating mitochondrial homeostasis, Regulating fatty acid metabolism	MASLD	(176)
Puerarin	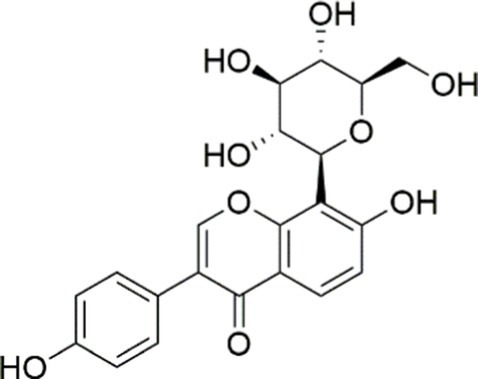	Activating the PI3K/AKT signaling pathway	Not assessed	HFD-induced C57BL/6 J mice, 200 mg/kg	Reducing ROS production	MASLD	(138)
Puerarin	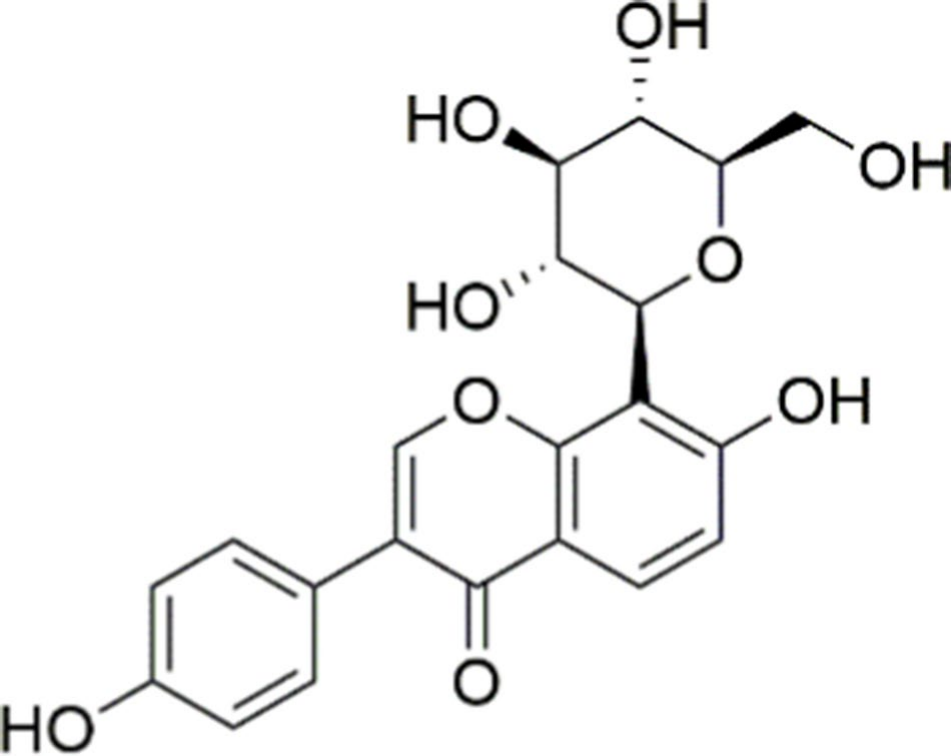	Activating the PI3K/AKT signaling pathway	FFA-induced HepG2 cells, 20, 40, and 80 μM; LPS and IFNCC/IFN-γ-induced RAW 264.7 cells, 40 and 80 μM	AMLN diets-induced C57BL/ 6 mice, 100 mg/kg; High-cholesterol diet-induced zebrafish, 100, 200, and 400 μg/mL	Ameliorating inflammatory responses	MASH	(139)
Liquiritigenin	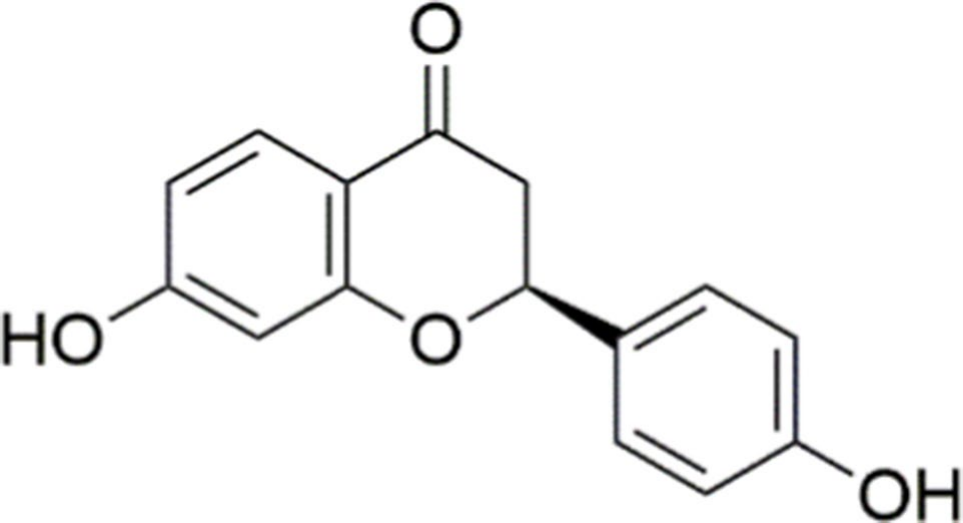	Activating the PI3K/AKT signaling pathway	Not assessed	HFD-induced C57BL/6 mice, 20, 40 mg/kg	Alleviating IR, Ameliorating inflammatory responses, Improving lipid accumulation	NASLD	(140)
Farrerol	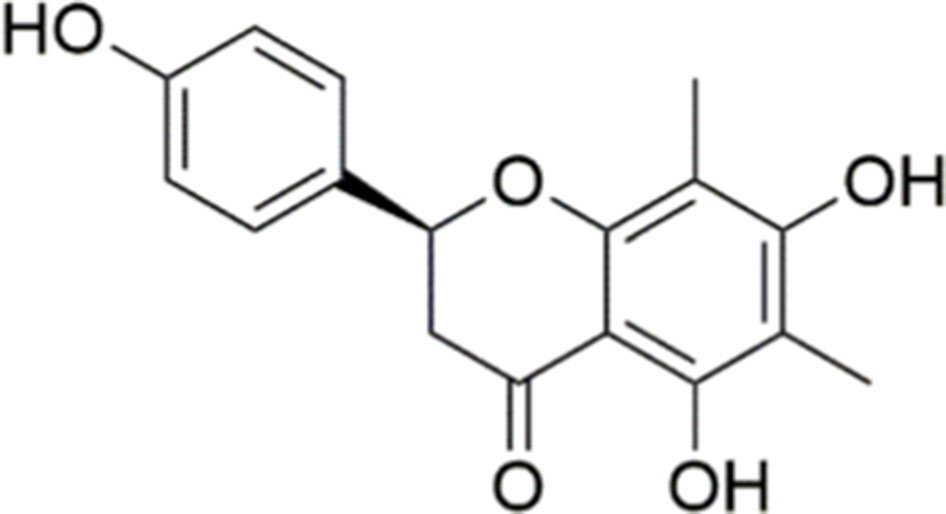	Activated PI3K/AKT signaling pathway	Not assessed	HFD-induced C57BL/6 J mice, 40 mg/kg	Alleviating IR, Inhibiting lipid accumulation	MASH	(141)
Didymin	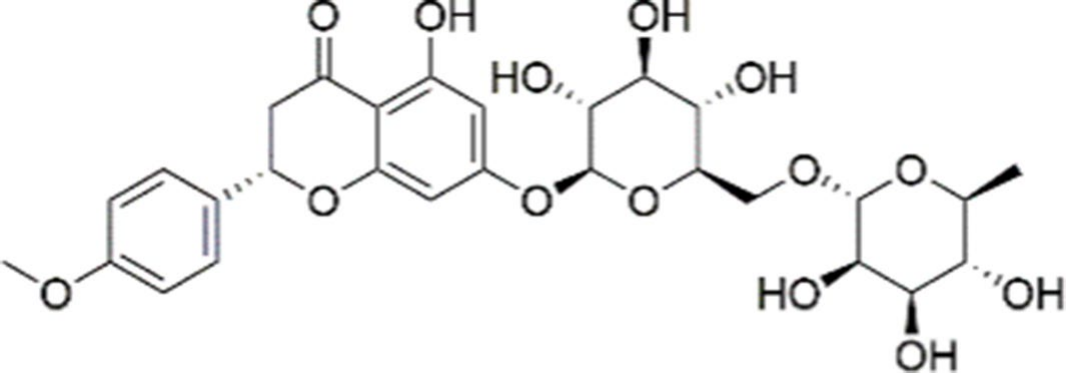	Inhibiting the PI3K/AKT signaling pathway	Not assessed	HFD and dexamethasone-induced C57BL/6 J mice, 0.4 and 0.8 mg/ kg	Ameliorating hepatic steatosis	MASLD	(142)
Hesperetin	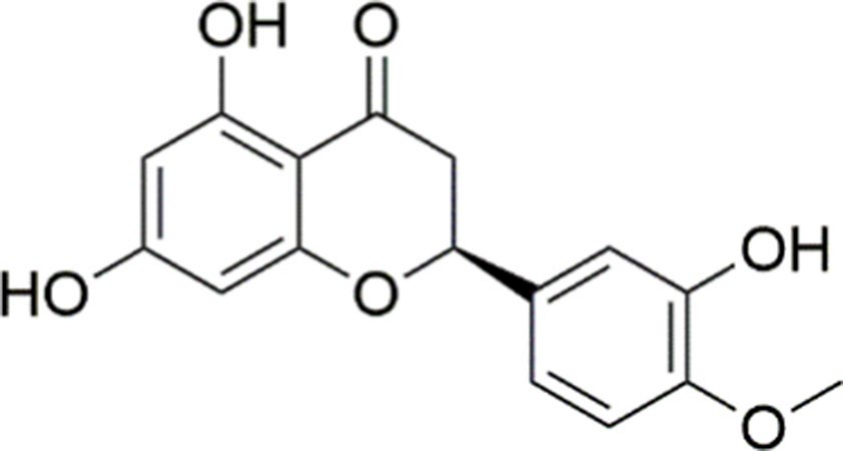	Activating PI3K/AKT-Nrf2-ARE signaling pathway	OA-induced HepG2 cells, 2.5, 5 or 10 μM	HFD-induced Wistar rats, 100, 300 mg/kg	Ameliorating hepatic oxidative stress, Ameliorating inflammatory responses	NASLD	(68)
Isoliquiritigenin	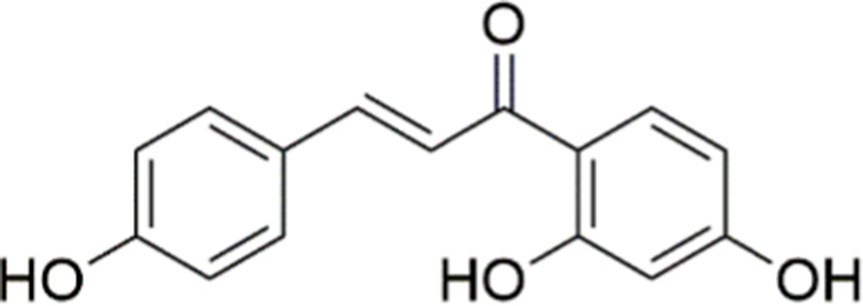	Inhibiting the PI3K/AKT/mTOR signaling pathway	PA induced AML12 cells, 10, 20 μM	CDAHFD-induced C57BL/6 mice, 10 mg/kg	Promoting autophagy	MASH	(143)
EGGG	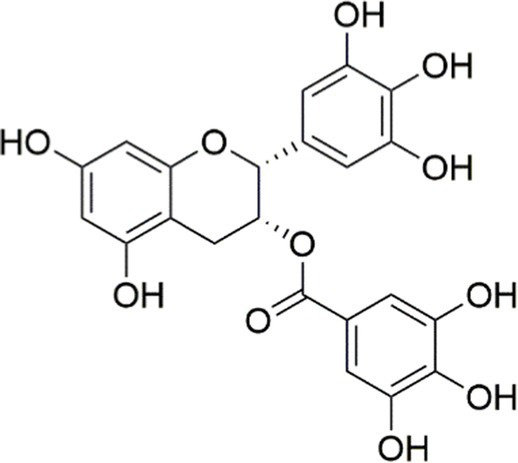	Activating the IRS1/IRS2/PI3K/AKT signaling pathway	Not assessed	HFD-induced SD rats, 0.32%EGCG in diets	Alleviating IR	MASLD	(144)
Kaempferol	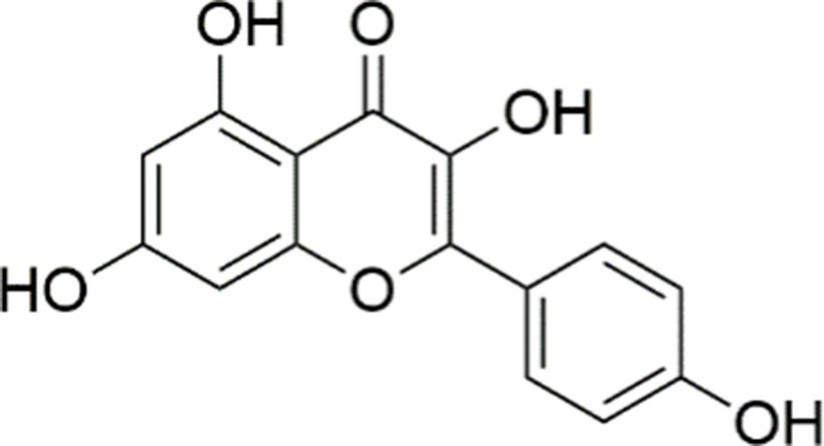	Activating the PI3K/AKT signaling pathway	OA-induced HepG2 cells, 10, 20 μM	Not assessed	Regulating lipid metabolism, Reducing apoptosis, Ameliorating inflammatory, Ameliorating autophagy	MASLD	(145)
Ginsenoside Re	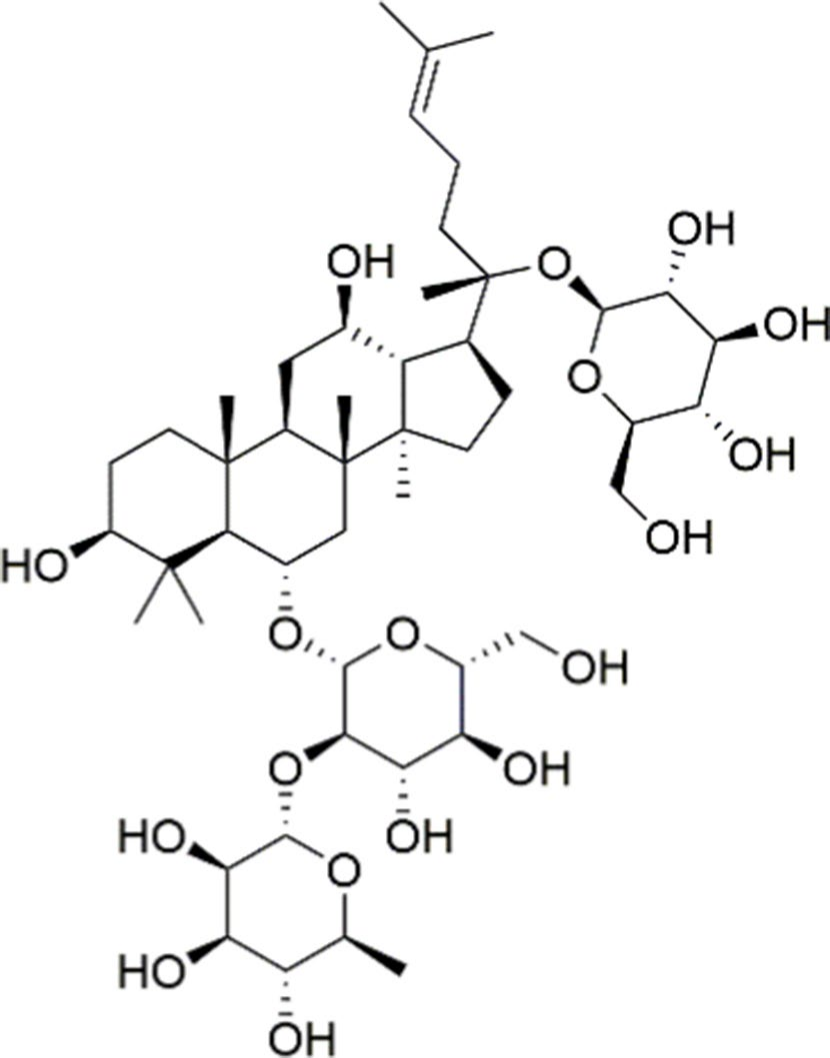	Activating the PI3K/AKT signaling pathway	Not assessed	HFD-induced C57BL/6 J mice, 20, 40 mg/kg	Regulating lipid metabolism	MASLD	(146)
Ginsenoside Rk3	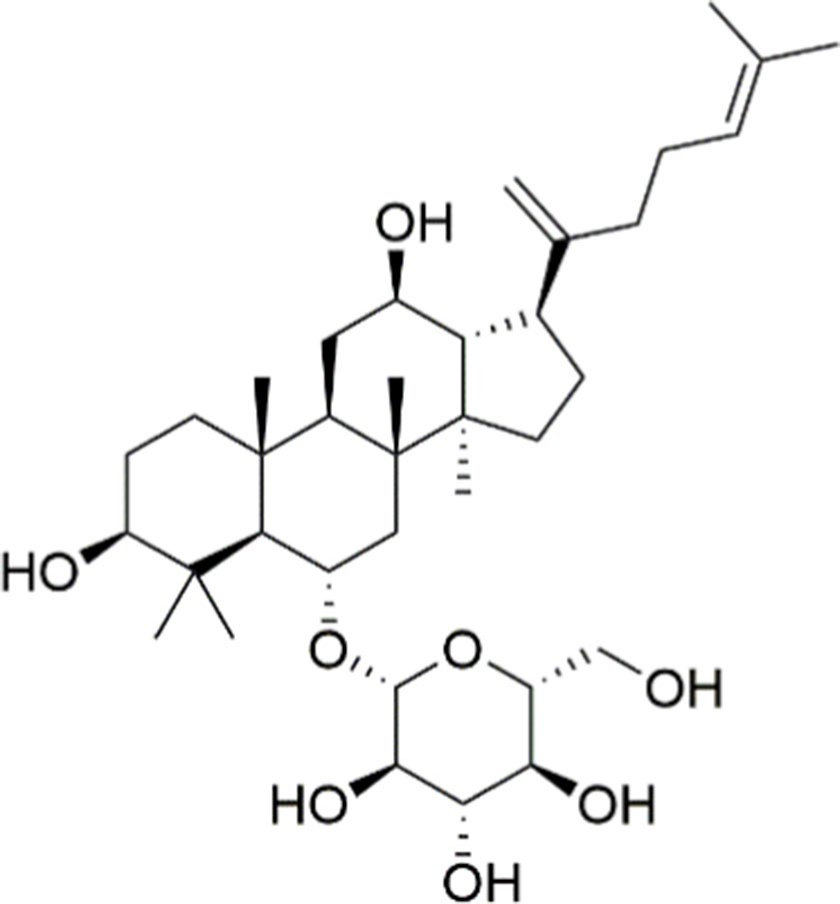	Activating the PI3K/AKT signaling pathway	PA and OA-induced HepG2 cells, 10, 20 μM	HFHCD and CCl4 injection-induced C57BL/6 mice, 60, 120 mg/kg	Regulating lipid metabolism	MASH	(147)
Calenduloside E	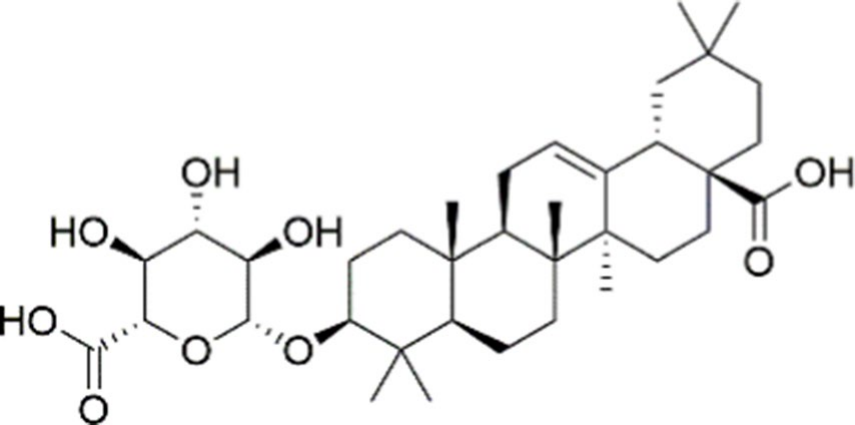	Inhibiting the PI3K/AKT/NF-*κ*B signaling pathway	A co-culture system using AML-12 and J774A.1 cells, 2, 4, 8 μM	Western diet-induced apoE^−/−^ mice, 5, 10 mg/kg	Ameliorating inflammatory responses	MASLD	(148)
Carnosic acid	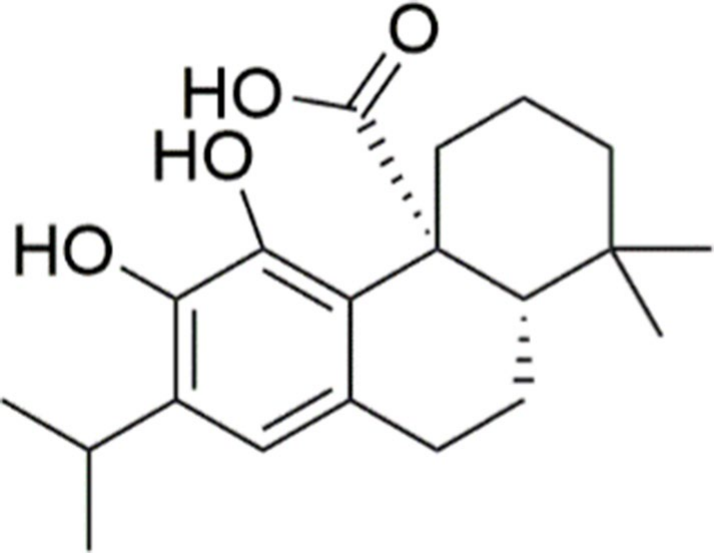	Inhibiting the PI3K/AKT signaling pathway	Not assessed	HFD-induced mice 15, 30 mg/kg	Ameliorating inflammatory responses, regulating lipid metabolism	MASLD	(149)
Sulforaphane	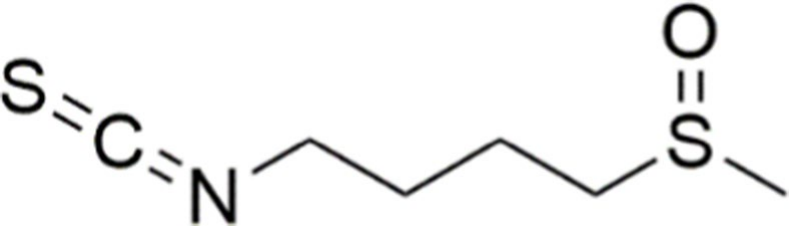	Activating the PI3K/AKT signaling pathway	Not assessed	HFD and ionizing irradiation-induced Wistar rats, 10 mg/kg	Alleviating IR, regulating lipid metabolism	MASLD	(15)
Lycorine	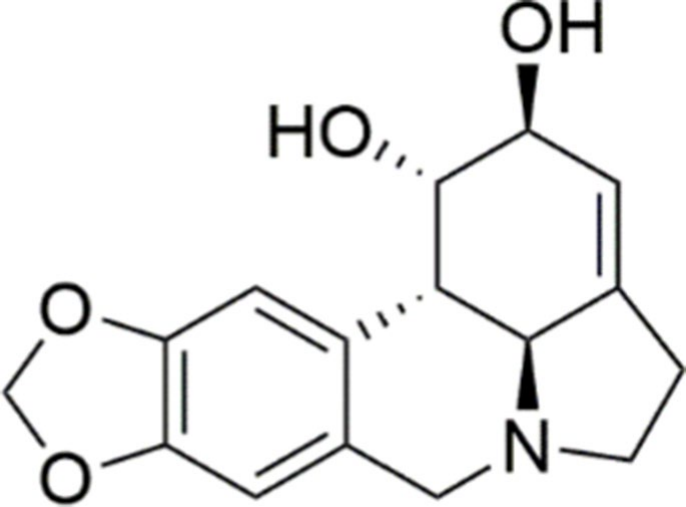	Inhibiting the PI3K/AKT signaling pathway	PA induced HepG2 cells, 0.5 μM	HFD-induced C57BL/6 J mice, 10, 20, 30 mg/kg	Ameliorating hepatic steatosis, oxidative stress and ferroptosis	MASLD	(151)
Diosgenin	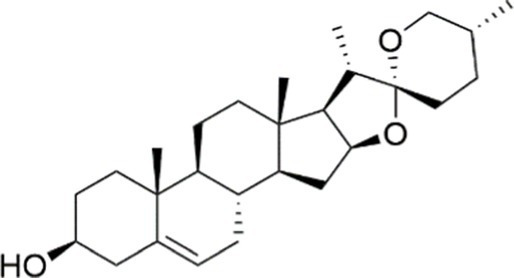	Activating the PI3K/AKT signaling pathway	FFA-induced HepG2 cells, 5,10, 25 μM	Not assessed	Regulating lipid metabolism, Ameliorating inflammation	MASH	(152)
Fucoidan	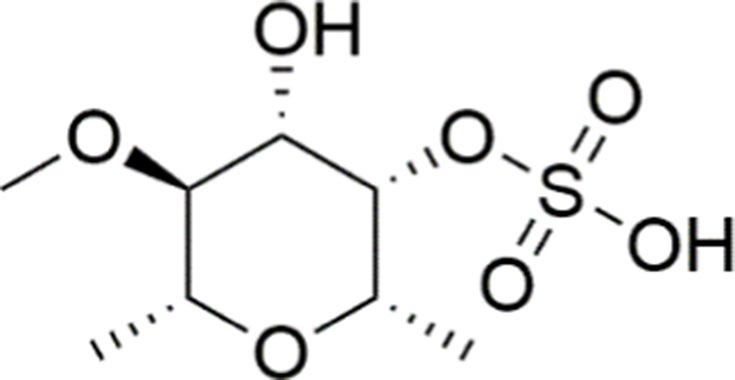	Activating the PI3K/AKT/ Nrf2 signaling pathway	FFA-induced Spheroids and HepG2 cells, 100 μg/mL	Not assessed	Reducing oxidative stress, Inhibiting inflammatory responses, Regulating lipid metabolism	MASLD	(67)
Combined curcumin and resveratrol	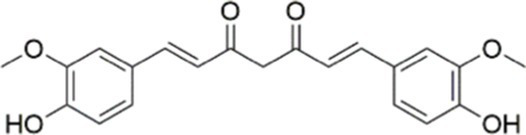 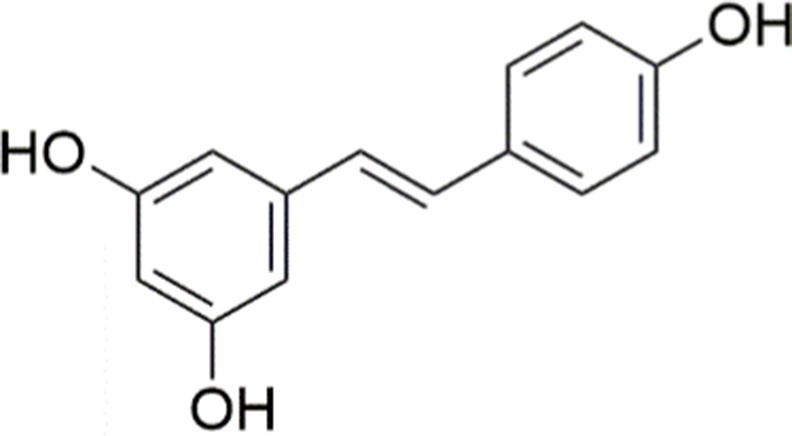	Inhibiting the PI3K/AKT/mTOR signaling pathway	PA-induced HepG2 cells, 2.5–10 μg/mL	HFD-induced Wistar rats and GK rats, 150 mg/kg	Regulating lipid metabolism	MASLD	(153)

### 3.2 Extracts from herbal medicines

#### 3.2.1 Herbal medicine formulas

In 2018, using PA-induced HepG2 cells and HFD-induced C57BL/6 J mice, Zheng *et al.* found that Jiang Zhi Granule (JZG), a clinically used herbal formula designed to treat patients with MASLD, could activate the autophagy process to protect against metabolic stress-induced hepatocyte injury in MASLD. And the potential mechanisms were correlated with the inhibition of the PI3K/AKT/mTOR signaling pathway (154). Similarly, Wang et al. found that the Modified Suanmei-Tang (MST) could also promote autophagy by suppressing phosphorylation in the PI3K/AKT/mTOR signaling pathway in the livers of HFD-induced MASLD mice (155). In 2024, Le et al. (156) evaluated the impact of Shenling Baizhu powder (SLBZ), a classic traditional Chinese medicine formula, on HFD-induced pregnant rats. The study revealed that SLBZ administration conferred a protective effect against MASLD in pregnant subjects. This hepatoprotective mechanism was suggested to involve the downregulation of key genes in the PI3K/AKT/mTOR signaling pathway, which played a critical role in regulating lipid homeostasis.

Based on the IRS/PI3K/AKT signaling pathway, researchers investigated four herbal medicine formulas named Gan-tang-yi decoction (GTYD), YiQi YangYin Decoction (YQ), Tangganjian decoction (TGJ) and Jiu Wei Qing Zhi Gao (JWQZG). Xu et al. (157) reported that GTYD could activate the IRS2/PI3K/AKT signaling pathway to reduce IR in MASH rats. Similarly, YQ was observed to promote the proliferation of pancreatic β-cells, an effect associated with the IRS-2/PI3K/AKT signaling pathway in a MASLD model (158, 159). Fan et al. (158) reported that treatment with TGJ led to a significant upregulation of IRS, PI3K, and AKT expression levels in a Wistar rat model induced by a HFHSD combined with streptozotocin. A separate investigation by Chen et al. (160) highlighted the therapeutic efficacy of JWQZG in the treatment of MASLD, demonstrating its regulatory influence on the insulin signaling pathway. The proposed molecular mechanism underlying this effect involved activation of the IRS1/PI3K/AKT/GSK3β signaling pathway.

In a 2019 study, the combined administration of ShengMai-Yin and Ganmaidazao decoction (SGD) was investigated in a mouse model of T2DM complicated with MASLD, revealing notable hepatoprotective effects. Researchers found that SGD could modulate liver glucose and lipid metabolism through co-activation of PI3K/AKT and PPAR*α* (161). Later, utilizing a minipig model of T2DM complicated with MASLD, Wang et al. (162) explored the underlying molecular mechanisms of Fufang Zhenzhu Tiaozhi capsule (FTZ). The results showed FTZ also regulated hepatic glycolipid metabolism through the PI3K/AKT signaling pathway. Xu et al. (4) examined the therapeutic efficacy of Shen-Shi-Jiang-Zhuo Formula (SSJZF) in an HFD-induced SD rat model. The findings indicated that SSJZF treatment resulted in a substantial downregulation of key lipogenic genes, including acetyl-CoA carboxylase (ACC, 5.3-fold), FAS (12.1-fold), SREBP1C (2.3-fold), and cluster of differentiation 3 (CD36, 4.4-fold), alongside a significant 67% reduction in collagen deposition. These effects were potentially mediated through the upregulation of p-PI3K/PI3K (1.6-fold) and p-AKT/AKT (1.6-fold) ratios (4) (Table 2, Figure 3).

**Table 2:** Protective effects of herbal medicine formulas on MASLD/MASH related to PI3K/AKT signaling pathway.

Agent	Compositions	Mechanism	In *vitro* activity/dose	*In vivo* activity/dose	Effects mediated by the PI3K/AKT signaling pathway	MASLD /MASH	Ref.
JZG	*Herba gynostemmatis*, *Radix salviae*, *Rhizoma polygoni cuspidati*, *Herba artemisiae scopariae*, *Folium nelumbinis*	Inhibiting PI3K/AKT/mTOR signaling pathway	PA-induced HepG2 cells, 100 μg/mL	HFD-induced C57BL/6 J mice, 994 mg/kg daily	Regulating autophagy	MASLD	(154)
MST	*Prunus mume*, *Crataegus pinnatifida Bunge*, *Citrus reticulata Blanco*, *Glycyrrhiza uralensis Fisch*	Inhibiting PI3K/AKT/mTOR signaling pathway	Not assessed	HFD-induced C57BL/6 J mice, 2.2, 1.1, 0.55 g/kg	Promoting autophagy, improving lipid metabolism	MASLD	(155)
SLBZ	Ginseng, *Atractylodes macrocephala*, *Poria cocos*, Yam, Semen Coicis, *Lentinus edodes*, Semen Armeniacae, *Platycodonis*, Lotus Root Meat, *Glycyrrhiza uralensis*	Inhibiting PI3K/AKT/mTOR signaling pathway	Not assessed	HFD-induced Sprague–Dawley rats, 1.6, 4.8 g/kg	Regulating lipid homeostasis	MASLD	(156)
GTYD	*Astragalus membranaceus* (Fisch.) Bunge, *Polygonatum sibiricum*, *Reynoutria japonica Houtt*, *Salvia miltiorrhiza* Bunge, *Cordyceps mycelium*, *Schisandra chinensis* (Turcz.) Baill, *Gynostemma pentaphyllum* (Thunb.) Makino, *Pinus massoniana* Lamb, Semen Persicae	Activating IRS2/PI3K/AKT pathway	Not assessed	CCl4 combined with HFHSD-induced SD rats, 10.6 g/kg/day	Increasing glycogen synthesis, mitigating IR	MASH	(157)
YQ	Radix Astragali, Chinese yam, Radix ginseng, *Polygonatum odoratum*, *Cornus officinalis*, Rhizoma polygonati, Radix Puerariae, Crataegi Fructus, Fermented soya beans	Activating IRS2/ PI3K/AKT signaling pathway	Not assessed	HFD and STZ-induced SD rats, 3.5, 7, 14 g/kg	Improving the growth of islet ß-cells, Ameliorating lipid accumulation	MASLD	(159)
TGJ	Radix Paeoniae Alba, *Angelica sinensis*, Oriental wormwood, *Polygonum cuspidatum*, *Schisandra chinensis*, *Poria cocos*, *Atractylodes*, Rhizoma Picrorhizae, Radix Bupleuri	Activating the IRS/PI3K/AKT signaling pathway	Not assessed	HFHSD and streptozotocin-induced Wistar rats, 0.44, 0.89, 1.77 g/mL	Improving glucose and lipid metabolism	MASLD	(158)
JWQZG	*Sedum sarmentosum* Bunge, *Schisandra chinensis* (Turcz.) Baill., *Paeonia lactiflora* Pall., *Lycopus lucidus* Turcz. ex Benth., *Poria cocos* (Schw.) Wolf, *Coix lacryma-jobi* var. *ma-yuen* (Rom. Caill.) Stapf, *Crataegus pinnatifida var. major*, *Coptis chinensis* Franch., *Faeces Bombycis*	Activating the IRS1/PI3K/AKT/GSK3*β* pathway	PA-induced HepG2 cells, Serum-containing JWQZG	HFD-induced C57BL/6 J mice, 1.85, 3.7, and 7.4 g/kg	Mitigating IR	MASLD	(160)
SGD	Radix Ginseng, Radix Ophiopogonis, Fructus Schisandrae; Radix Glycyrrhizae, Fructus Tritici levis, Fructus Zizyphi Jujubae	Activating the PI3K/AKT signaling pathway	Not assessed	HFD-induced KKAy mice, 5.0, 10, 15 g/kg	Suppressing fatty acid synthesis, Increasing insulin sensitivity	MASLD	(161)
FTZ	*Ligustrum lucidum W. T. Aiton*, *Coptis chinensis* Franch., *Salvia Miltiorrhiza* Bunge., *Panax notoginseng* (Burkill) F.H. Chen., *Cirsium japonicum* (Thunb.) Fisch. Ex DC., *Eucommia ulmoides* Oliv, *Citrus medica* L., *Atractylodes macrocephala* Koidz	Activating the PI3K/AKT signaling pathway	Not assessed	HFD and streptozotocin-induced Chinese Wuzhishan minipigs, 1.2 g/ kg	Improving glucose and lipid metabolism	MASLD	(162)
SSJZF	*Atractylodes lancea*, *Poria cocos*, *Magnolia officinalis*, *Rhizoma Pinelliae*, Talcum, *Tetrapanax papyriferus*, *Radix Bupleuri*, *Citrus aurantium* L., Radix paeoniae rubra, Panax notoginseng, *Rubia cordifolia* Linn, *Fructus Broussonetiae*, *Pinellia ternate*	Activating the PI3K/AKT signaling pathway	Not assessed	HFD-induced SD rats 50, 100 and 200 mg/kg	Regulating lipid metabolism	NASLD	(177)

#### 3.2.2 Plant extracts

Artemisia capillaris (AC) is commonly used in traditional medicine with various pharmacological activities. Liang *et al.* demonstrated that the aqueous extracts of AC (WAC) activated the PI3K/AKT signaling pathway in both *in vivo* and *in vitro* MASLD models. This activation was associated with enhanced AMPK phosphorylation, leading to reduced SREBP-1c protein expression, suppression of fatty acid synthesis, and decreased TG content (163). *Polygonum perfoliatum* L. (PPL) is classified as a traditional Chinese herbal medicine historically employed for the treatment of thoracic fullness. By integrating network pharmacology predictions with experimental validation, Liu et al. (164) identified the therapeutic potential of PPL against MASLD. They subsequently confirmed that its protective effect involved the dual activation of the PI3K/AKT-mediated glucolipid metabolism pathway and the hepatic NF-*κ*B-mediated cytokine signaling pathway. Niu et al. (69) investigated the protective effects of pink lotus essential oil (PLEO) on MASLD. Their study was the first to demonstrate that PLEO attenuated FFA-induced steatosis in HepG2 cells by regulating lipid metabolism, suppressing inflammatory responses, and improving IR. Furthermore, PLEO inhibited the secretion of TNF-α, IL-6, and IL-1β, reduced the expression of NF-*κ*B, FAS, ACC, and SCD1, while increasing the phosphorylation levels of PI3K, AKT, and carnitine palmitoyltransferase-1 (CPT-1).

Wu et al. (70) reported that Corydalis saxicola Bunting Total Alkaloids (CSBTA) could prevent the onset of MASH in mice, reduce intrahepatocellular lipid accumulation, and suppress hepatocyte inflammation under HFD conditions. Mechanistic studies revealed that these protective effects were mediated by regulating the PI3K signaling pathway and TLR4/NF-*κ*B pathways (70). Another total alkaloid was extracted from *Rhizoma Coptidis*. To investigate the efficacy and mechanism of total *Rhizoma Coptidis* alkaloids (TRCA) against MASH, researchers employed an HFD-induced mouse model. The study demonstrated that TRCA alleviated HFD-induced liver injury by rescuing the suppressed PI3K/AKT signaling pathway. This finding was consistent across *in vitro* models, where TRCA treatment markedly increased the diminished levels of p-PI3K and p-AKT in FFA-induced HepG2 and LO2 cells (165).

In 2021, Niu et al. (166) reported that Corni Fructus iridoid glycosides exerted therapeutic effects on a mouse model of T2DM combined with MASLD. The effects were achieved by activating the PI3K/AKT pathway to ameliorate T2DM and inhibiting the NF-*κ*B pathway to mitigate MASLD. Based on the HFD-induced MASLD mouse model, researchers found that both *Platycodon grandiflorus* and the Triterpenic acids-enriched fraction from *Cyclocarya paliurus* (CPT) could attenuate IR *via* the PI3K/AKT/GSK3β signaling pathway (10, 167). Hwang et al. (168) investigated the effects of extracts from *Aralia elata* (Miq.) Seem. (AE extracts) on OA-induced HepG2 cells and HFD-induced C57BL/6 J mice. The study revealed that AE extracts exerted their beneficial effect against MASLD by means of activating the PI3K/AKT/GLUT4 pathway, thereby counteracting IR. *Psoralea corylifolia* L. (PC) is a traditional Chinese herb historically used in the treatment of pediatric diseases characterized by yang deficiency of the spleen and kidney. In 2017, Zhou et al. (9) reported that PC concentrated granules alleviated hepatic steatosis, inflammatory cell infiltration, and fibrosis in juvenile MASLD/MASH mice. Interestingly, PC treatment suppressed the hepatic expression of PI3K p85 protein and diminished the p-AKT/total AKT ratio. This downregulation, rather than an IR-related effect, appeared to be a key pathway through which PC exerted its anti-fibrotic action (9).

Pueraria flavonoids, one of the main active constituents of *Pueraria lobata*, were found by Sun et al. (97) to effectively reduce hepatic lipid deposition and inflammatory damage. Further studies revealed that the therapeutic effect was achieved through the inhibition of the PI3K/AKT/mTOR signaling pathway and the concurrent activation of autophagy, consequently ameliorating intracellular lipid accumulation and inflammation. Wu et al. (126) demonstrated in another study that pure total flavonoids from *Citrus* (PTFC) ameliorated MASH symptoms. They proposed that this effect was potentially mediated by regulating the CCL2/CCR2-PI3K-AKT signaling pathway to reduce hepatic inflammatory responses (Table 3, Figure 3).

**Table 3:** Protective effects of plant extracts on MASLD/ MASH related to PI3K/AKT signaling pathway.

Agent	Mechanism	In *vitro* activity/dose	In *vivo* activity/dose	Effects mediated by the PI3K/AKT signaling pathway	MASLD/MASH	Ref.
WAC	Activating the PI3K/AKT signaling pathway	OA-induced HepG2 cells, 1.5 mg/mL	HFD-induced C57BL/6 J mice, 50 mg/kg	Reducing lipid synthesis	MASLD	(163)
PPL	Activating the PI3K/AKT signaling pathway	PA, OA and high glucose-induced AML12 cells, 5, 10 μg/mL	HFD-induced C57BL/6 mice, 200, 400 mg/kg	Improving glucose and lipid metabolism	MASLD	(164)
PLEO	Activating the PI3K/AKT signaling pathway	OA and PA-induced HepG2 cells, 0.01, 0.1, 1 μg/mL	Not assessed	Alleviating IR, Regulating lipid metabolism, Inhibiting the inflammatory response	MASLD	(69)
CSBTA	Activating the PI3K/AKT signaling pathway	PA OA and LPS-induced HepG2 cells, 0, 12.5, 25 and 50 μg/mL	HFD with high carbohydrate drinking-induced C57BL/6 J mice, 25, 50, 100 mg/kg	Attenuating inflammation, attenuating hepatic lipid accumulation	MASH	(70)
TRCA	Activating the PI3K/AKT signaling pathway	FFA-induced LO2and HepG2 cells, 40, 80, 120 μg/mL	HFD-induced C57BL/6 J mice, 37.5, 75, 150 mg/kg	Ameliorating lipid accumulation ameliorating inflammatory responses	MASH	(165)
Iridoid Glycoside from Corni Fructus	Activating the PI3K/AKT signaling pathway	Not assessed	HFHS diet and STZ-induced ICR mice, 200, 300, and 400 mg/kg	Improving IR	MASLD	(166)
*Platycodon grandiflorus*	Activating PI3K/AKT/GSK3*β* signaling pathway	Not assessed	HFD-induced C57BL/6 J mice, 2 g/kg	Improving IR	MASLD	(10)
Triterpenic acids-enriched fraction from CPT	Activating PI3K/AKT/GSK3*β* signaling pathway	PA-induced HepG2 cells, 25 μg/mL	HFD-induced C57BL/6 J mice, 40, 160 mg/kg	Improving IR	MASLD	(167)
AE extracts	Activating PI3K/AKT/GLUT4 signaling pathway	OA-induced HepG2 cells, 100 μg/mL	HFD-induced C57BL/6 J mice, 100, 300 mg/kg	Improving IR	MASLD	(168)
PC	Inhibiting PI3K/AKT signaling pathway	Not assessed	HFD-induced C57BL6J mice, 1.125, 2.25 mg/g	Attenuating fibrosis	MASLD/MASH	(9)
Pueraria flavonoids	Inhibiting PI3K/AKT/mTOR signaling pathway	OA and PA-induced HepG2 cells, 12.5, 25, 50 μg/mL	HFD-induced C57BL/6 J mice, 50, 100 and 200 mg/kg	Activating autophagy, reducing lipid deposition, attenuating inflammation	NASLD	(97)
PTFC	Inhibiting CCL2/CCR2-PI3K/AKT signaling pathway	Not assessed	HFD-induced C57BL/6 mice, 50 mg/kg	Attenuating inflammatory	MASH	(61)

## 4 Conclusions and future directions

MASLD and its progressive form, MASH, pose a significant threat to the quality of life and safety of hundreds of millions of patients globally, representing a common and pressing worldwide health challenge. Hence, it remains imperative to further elucidate the underlying pathophysiology of these conditions and develop targeted interventions based on their molecular mechanisms. The modulation of cell signaling pathways has become a prominent strategy in pharmaceutical development. Natural products have gained significant attention as an investigational focus, valued for their prospect of offering greater efficacy and reduced toxicity. In the present paper, we summarize the involvement of the PI3K/AKT signaling pathway and its downstream effectors in the progression of MASLD/MASH, with a particular focus on the preventive potential of natural monomers and extracts derived from herbal medicines that target this pathway. Research continues to advance our knowledge of how natural agents like phenylpropanoids, flavonoids, terpenoids, and alkaloids protect against MASLD/MASH *via* the PI3K/AKT signaling pathway. Nevertheless, their drug development potential awaits validation through detailed preclinical and clinical studies.

First, the animal models of some agents mentioned in our paper are lacking, particularly concerning MASLD/MASH treatment. It is thus recommended to translate *in vitro* results into *in vivo* models. Furthermore, the ultimate goal of “bench-to-bedside” translation must be emphasized, a process that hinges on validation through rigorous randomized controlled trials, which represent the gold standard for evaluating the therapeutic efficacy of herbal-derived compounds. Consequently, in-depth mechanistic exploration and further clinical validation emerge as the two pivotal steps in future development.

Secondly, given that MASLD/MASH typically necessitates prolonged treatment, oral medications remain the preferred therapeutic option. However, limited oral bioavailability remains a primary constraint for the majority of these agents, as exemplified by curcumin and Ginsenoside Rk3. Both curcumin and ginsenoside Rg3 exhibit low oral bioavailability in rats (<1 and 2.63%, respectively) due to their poor solubility in water. And their oral bioavailability is similarly low in humans (169, 170). Silymarin, which has been identified for clinical use as a treatment for liver disease, has also faced the same problem in the past (171). Studies have reported an absolute oral bioavailability of approximately 0.95% for silibinin in rats (172). Various strategies have been studied to improve the dissolution and bioavailability of silibinin, and promising results have been achieved (173). Similarly, several methods have been explored to improve the bioavailability of curcumin and ginsenosides. These include encapsulating them into micelles, liposomes, emulsions, and other nano-sized delivery systems to enhance intestinal absorption (174, 175). Thus, a key future direction involves optimizing the bioavailability of promising anti- MASLD/MASH drug candidates, especially for those targeting the PI3K/AKT signaling pathway. To this end, the development of advanced drug delivery systems and novel formulations is essential for these compounds. Furthermore, it is imperative to systematically investigate the structure–activity relationships of these compounds. Such research will inform targeted structural modifications to enhance efficacy and establish a theoretical basis for rational drug development.

Thirdly, the development of these natural agents is limited by insufficient pharmacokinetic data and a scarcity of toxicity studies. Future work must therefore prioritize comprehensive PK profiling and systematic evaluations of adverse effects. Last, the safety profiles of the metabolites generated from natural products have been largely neglected in current research and require urgent investigation. It is particularly notable that metabolites with defined structures, such as dihydrocurcumin and tetrahydrocurcumin derived from curcumin, exhibit significant biological activity, thereby confirming their direct contribution to the efficacy of natural products. A critical focus lies on the structural characteristics of natural product metabolites, given their determining role in intestinal absorption and consequent pharmacological activity.

Despite the necessity for deeper research, monomers and extracts isolated from herbal medicines exhibited potential as treatments for MASLD/MASH.
